# Methods of Lysergic Acid Synthesis—The Key Ergot Alkaloid

**DOI:** 10.3390/molecules27217322

**Published:** 2022-10-28

**Authors:** Michał K. Jastrzębski, Agnieszka A. Kaczor, Tomasz M. Wróbel

**Affiliations:** 1 Department of Synthesis and Chemical Technology of Pharmaceutical Substances with Computer Modeling Laboratory, Faculty of Pharmacy, Medical University of Lublin, 4A Chodźki St., PL-20093 Lublin, Poland; 2 School of Pharmacy, University of Eastern Finland, Yliopistonranta 1, P.O. Box 1627, FI-70211 Kuopio, Finland; 3 Department of Drug Design and Pharmacology, Faculty of Health and Medical Sciences, University of Copenhagen, Universitetsparken 2, 2100 Copenhagen, Denmark

**Keywords:** lysergic acid, ergot alkaloids, total synthesis, biosynthesis

## Abstract

Ergot is the spore form of the fungus Claviceps purpurea. Ergot alkaloids are indole compounds that are biosynthetically derived from L-tryptophan and represent the largest group of fungal nitrogen metabolites found in nature. The common part of ergot alkaloids is lysergic acid. This review shows the importance of lysergic acid as a representative of ergot alkaloids. The subject of ergot and its alkaloids is presented, with a particular focus on lysergic acid. All methods of total lysergic acid synthesis—through Woodward, Hendrickson, and Szantay intermediates and Heck coupling methods—are presented. The topic of biosynthesis is also discussed.

## 1. Lysergic Acid as a Component of Ergot Alkaloids

### 1.1. Ergot

Ergot is the spore form of the fungus *Claviceps purpurea* (Fr.) Tul. (see Section Abbreviations used in the text). It occurs in the form of elongated sclerotia, ranging in length from a few millimeters to 4–6 cm. Ergot, in addition to causing diseases of cereal and grass, and, hence, lowering yields, also causes grain contamination with toxic substances, alkaloids, which are harmful to both humans and animals, causing a number of diseases commonly referred to as “ergotism” [1].

#### 1.1.1. History of Ergot [2]

The problem of the presence of ergot and its toxicity has existed since the agrarian revolution. There is historical evidence of their presence in numerous cultures, ranging from the Assyrians (600 BC), through ancient Rome (1st century BC), to medieval Europe. Typically, ergot has been described as “harmful spots on the spikes” and “poisonous grass causing miscarriage”. Hippocrates identified a plant disease in the 4th century BC, which he called *melanthion*—and described the inhibitory effect of ergot on postpartum bleeding [3].

Ergotic epidemics frequently occurred in medieval Europe. They were due to the prevalence of contaminated rye used in baking bread. Rye often contained large amounts of alkaloids that induced ergotism. The very first ergot epidemic was mentioned in *Annales Xantensen* (Germany, 857). Numerous sources describe successive epidemics in France, Germany, and Scandinavia. The 16th century was particularly dramatic, when tens of thousands of people died in Europe from ergot poisoning. The last major epidemic took place in Russia in 1926–1927 in the Ural region. Single cases of ergotamine poisoning have occurred since then [1,4].

One of the first to make connection between illness and eating contaminated grain was the German physician Wendelin Thelius, in 1596. In 1670, Thuillier linked the presence of ergot in rye bread with epidemics by experimenting with feeding ergot to animals, which led to their death. At the end of the 18th century, L’Abbe Tessier proposed preventive measures—compulsory grain cleaning and replacing contaminated grain with potatoes in the diet. It was not until 1764 that von Münchhausen stated that ergot was a fungus [1].

Ergot was also used for medicinal purposes. Ergot alkaloids were used as early as 2000 BC in Greece as a hallucinatory agent, in Mesopotamia as an ingredient of “magic spells”, and in Egypt in a formulation described as a hair growth remedy. The first confirmed reports of the deliberate use of ergot come from around 1100 B.C.E. from China, where it was used in obstetrics and gynecology. In the 16th century, ergot was also used for medical purposes in the West—Adam Lonicer used it to induce labor, and Thalius used ergot to stanch the bleeding. In 1820, ergot was introduced into the *United States Pharmacopoeia* [1].

Nowadays, the presence of ergot in cereal is no longer a problem. Grain cleaning techniques used in mills remove up to 82% of the ergot contained in them. In addition, the process of baking wheat bread allows for the reduction of the concentration of alkaloids, even to zero [4].

#### 1.1.2. Properties of Ergot

As described above, ergot is both toxic and therapeutic [4]. Two different forms of ergot poisoning have been described: ergotismus gangrenosus (gangrene, “St. Anthony’s fire”) and ergotismus convulsivus (contractile form, “St. Vitus dance”). The two forms can coexist or be independent of each other. Symptoms present in gangrenous form, include diarrhea, sensory disturbances, slow heart rate, shortness of breath, paralysis of the respiratory center, ischemia affecting the limbs, thrombotic changes followed by gangrene, loss of limbs, and death. In the case of the systolic form, symptoms comprise of sensory disturbances, muscle tremors, headache, abdominal pain, slow heart rate, and, at the same time, strong nervous excitation [3,4].

Ergot is also toxic to animals, causing symptoms similar to those observed in humans: loss of milk, convulsive ergotism, and gangrene.

The therapeutic effects of ergot may include inhibition of prolactin, treatment of parkinsonism [5], treatment of cerebrovascular insufficiency, migraine, venous insufficiency, thrombosis, embolism, stimulation of cerebral, peripheral metabolism, and stimulation of the uterus [1,4].

Moreover, ergot alkaloids have been used to induce abortion for hundreds of years [3].

### 1.2. Ergot Alkaloids

#### 1.2.1. Types of Ergot Alkaloids

**Figure 1:**
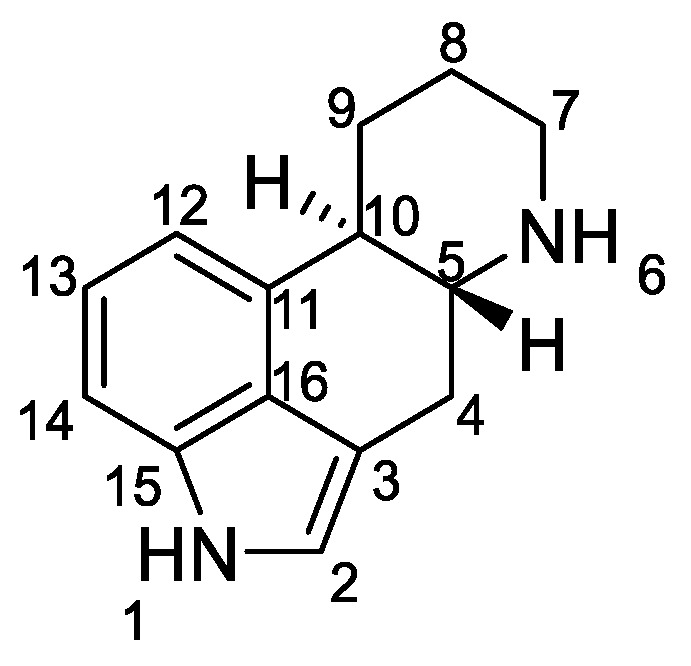
Structure of ergoline.

Ergot alkaloids are based on the ergoline ring system (Figure 1).

Ergoline derivatives have two asymmetric centers, either at the C-5 and C-10 positions or at the C-5 and C-8 positions. The stereochemistry and configuration of all atoms in the structures are accurately described in the literature, in particular the relative positions of the hydrogen [6,7].

Ergot alkaloids are indole compounds that are biosynthetically derived from L-tryptophan and represents the largest group of fungal nitrogen metabolites found in nature. Ergot contains 0.15% to 0.5% of alkaloids, which can be separated by analytical methods into individual ones, with more than 80 individual ergot alkaloids [2].

**Figure 2:**
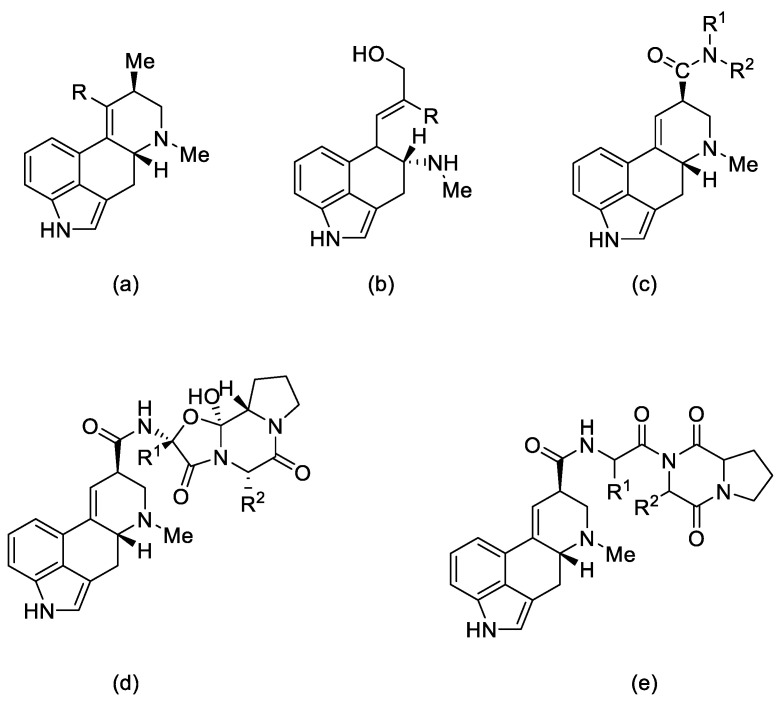
Structural formulas of the 5 main groups of ergot alkaloids: (**a**) clavine alkaloids and (**b**) 6,7-secoergolenes (alkaloids with an open D ring), (**c**) lysergic acid derivatives, (**d**) peptide alkaloids, and (**e**) lactam ergot alkaloids.

There are five main groups depending on the R substituent in the C-8 position (Figure 2) [8,9].

Ergot alkaloids can also be classified according to their molecular weight. Low molecular weight ergot alkaloids are amides of lysergic (or isolysergic) acid and amino alcohols. Ergometrine (ergobasine, ergonovine, ergoklinine, ergostetrine, and ergotocine) is an amide of 2-aminopropanol and lysergic acid, and ergometrinine (ergobasinine and isoergometrine) is an amide of isolysergic acid. High molecular weight ergot alkaloids are oligopeptides consisting of a ring of three amino acids (always with proline), linked to lysergic acid by a peptide bond. These are compounds such as: ergotamine, ergotin, ergosine, ergotoxin, ergocryptine, ergocristine, ergoclavine, and chanoclavine [1].

#### 1.2.2. Biochemical and Pharmacological Properties

**Figure 3:**
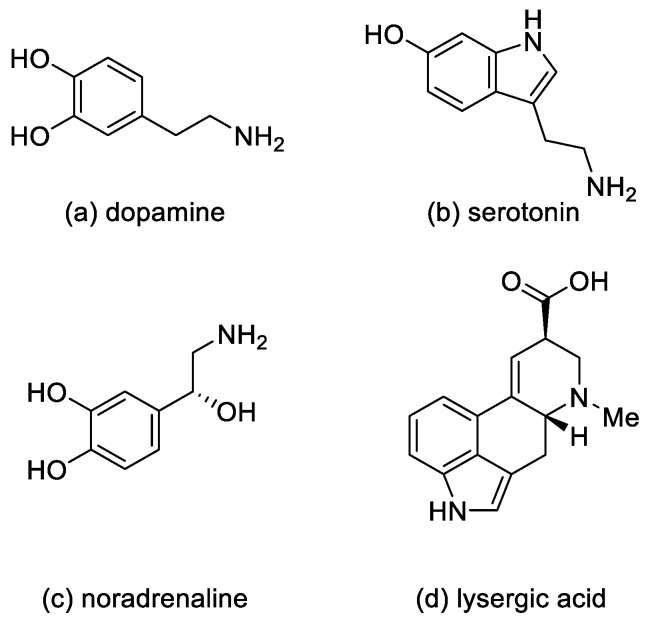
Structural formulas of endogenous amines and lysergic acid.

Ergot alkaloids—derivatives of D-lysergic acid—act by antagonistic or agonistic affinity to α adrenergic receptors as well as to serotonin receptors and dopamine receptors (D_1_–D_5_) [10]. This is due to the structural similarity to endogenous amines—noradrenaline, dopamine [11], and serotonin [12] (Figure 3).

In medicine, ergotamine, dihydroergotamine, dihydroergotoxin, and dihydroergocristine, as well as their mixtures, are used [10].

These compounds are applied in medicine as sedatives and painkillers. They are also used as antispasmodics (in migraine attacks and in gynecology, in particular ergotamine); as drugs in orthostatic hypotension, in the treatment of intellectual disorders, and after strokes (for example, dihydroergotoxins) [10].

**Figure 4:**
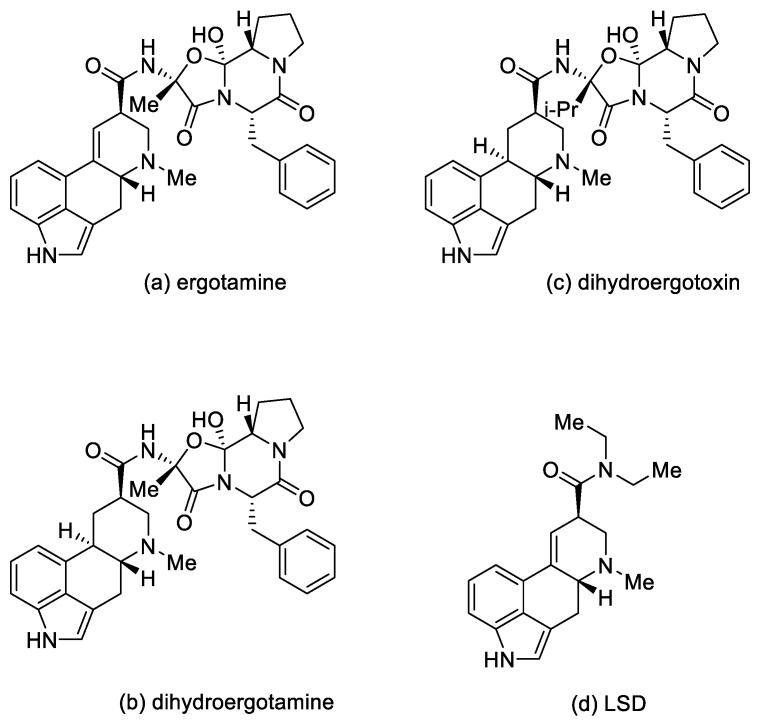
Ergot alkaloid derivatives.

Lysergic acid diethylamide (LSD) (Figure 4) is a semisynthetic hallucinogenic compound. It is one of the most active psychedelic substances with hallucinogenic effects—the mild effects appear at a dose of 25 µg. LSD is an agonist of the 5-HT_2A_ receptor located on the inhibitory interneurons of the brain. However, the action of LSD is not limited to that of the serotonin receptors. Although LSD and the other psychoactive derivatives exert their effects through activation of 5-HT_2A_ receptors, they can have a significant behavioral component mediated by activation of 5-HT_1A_ receptors [13,14]. Moreover, LSD also has an effect on the dopamine D_1_ and D_2_ receptors [15].

Due to a short-term reduction in serotonin levels, after the LSD interaction with cells subsides, its production in the cells increases abruptly, which may cause disintegration of cortical–cortical neuronal activity—and, consequently, induce increased perception of sensory stimuli and cognitive impairment [16]. Interestingly, LSD is not addictive, and despite the fact that it is not currently used in medicine, there are ongoing studies on its use in psychiatry—e.g., in the fight against alcoholism [17,18].

Ergot alkaloids cause effects such as vasoconstriction, uterine contraction, and ergotism of varying intensity (Table 1) [19].

#### 1.2.3. Other Application [20]

**Figure 5:**
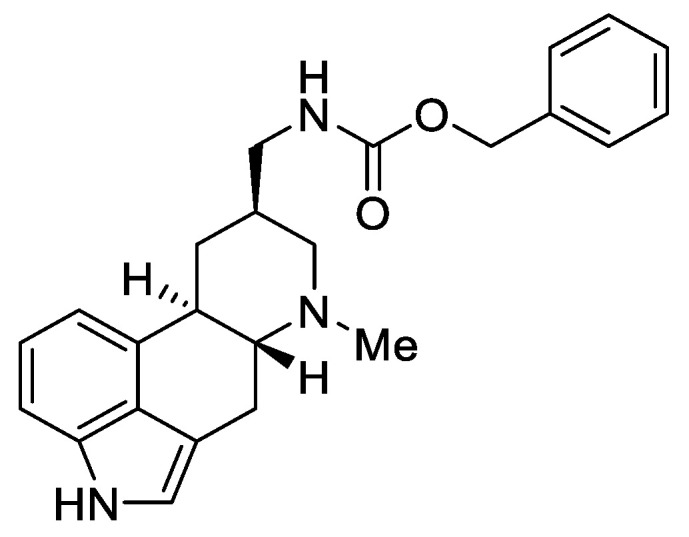
Metergoline.

According to the latest reports, a semisynthetic lysergic acid derivative containing a urethane bond at the C-8 substituent—metergoline (Figure 5), has an interesting antibacterial application.

This compound is authorized under the trade names Contralac and Liserdol as a hyperprolactinemia drug [21]. Previous studies have indicated its agonistic activity towards the 5 HT1A, 5-HT1D, and 5-HT1B receptors, as well as antagonistic activity towards the 5-HT_2A_, 5-HT_2B_, 5-HT_2C_, and 5-HT_7_ receptors [22,23].

The latest data, published in 2022 by Brown and Magolan [20], indicate that metergoline is an inhibitor of the intracellular Gram-negative pathogen *Salmonella typhimurium*. The biomechanism of action assumes a change in the permeability of the outer membrane of SPR741, which allows for an increase in the potential of biological activity against the MRSA strains, *WT E. coli*, *Acinetobacter baumannii*, and *Burkholderia cenocepacia*. The authors suggest that the ergot scaffold (especially the amide derivatives of lysergic acid, such as metergoline) may be the basis for the production of new efficient antibiotics.

### 1.3. Lysergic Acid

**Figure 6:**
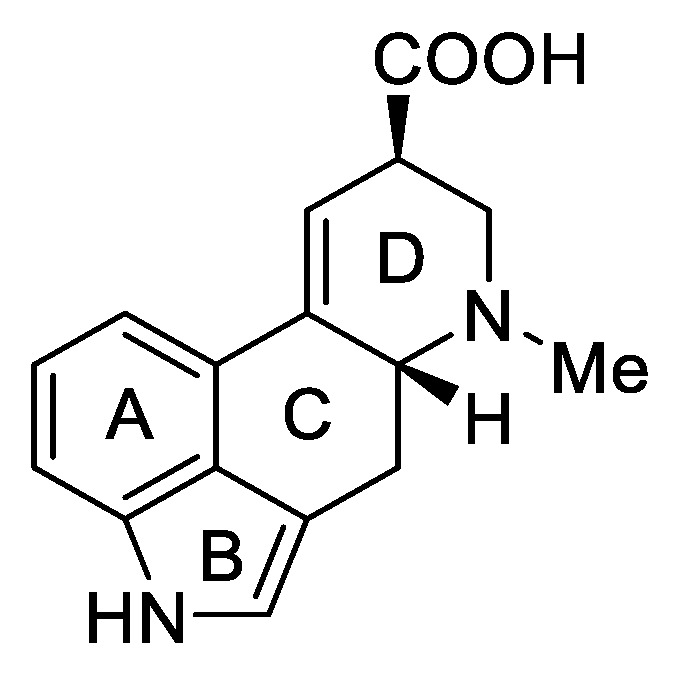
*d*-Lysergic acid.

Lysergic acid (Figure 6) is a chemical compound based on the structure of ergoline—a four-ring system—which has a COOH acid group in the C-8 position and a methylamine in the 6-position in the D ring.

Lysergic acid is a very important chemical compound obtained on a large scale. This is because it is a key precursor for the preparation of more complex ergot alkaloids, especially lysergic acid amides.

#### History of Lysergic Acid [24]

The isolation and analysis of ergot alkaloids began in 1906, when ergotoxine (a mixture of three proline-based alkaloids, ergocristine, ergocriptine, and ergocornine) was isolated from ergot [25], and its adrenolytic activity was discovered [26]. Twelve years later, Stoll isolated ergotamine—the first chemically pure ergot alkaloid, with major medical uses [27]. From that moment, the research efforts intensified. Since 1934, comprehensive studies of ergot alkaloids have been carried out by way of collaboration between teams in the USA, England, and Switzerland, with pharmaceutical companies investigating the pharmacological and clinical effects [28]. As a result, Jacobs and Craig identified the common part for ergot alkaloids—lysergic acid [29].

**Figure 7:**
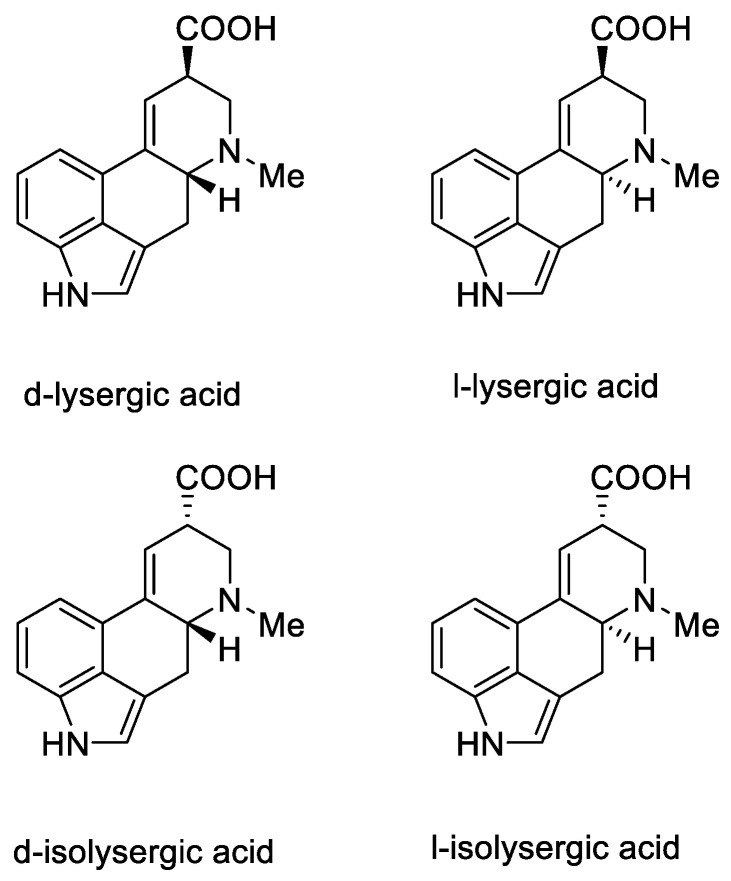
Four stereoisomeric forms of lysergic acid.

Lysergic acid is a chiral compound with two stereogenic centers. Subsequently, it was possible to identify all four stereoisomeric forms in which this acid can occur: *d*-lysergic acid, *l*-lysergic acid, *d*-isolysergic acid, and *l*-isolysergic acid; this has been described by Stoll, Hofmann, and Troxler (Figure 7) [30].

Finally, in 1956, at the Lilly Laboratory, the Woodward group was the first to achieve a total synthesis of lysergic acid [31]. In the following years, further structures of ergot alkaloids were identified, and the total syntheses of ergotoxine components were presented.

An explanation of the proposed structures was summarized in the synthetic works of Woodward [31] and Ramage [32]. By examining the pentacyclic styrene derivatives as well as the 2,3-dihydrolysergic acid (+)-butanolamide in the lysergic acid derivatives [33], a conclusion was made about the C-H bonds at C-3 and C-5 being cis and quasi-axial [34].

Surprisingly, not only is the carboxyl/ester group at the C8 position stereoselectively labile, but there is lability at both chiral centers, including at C5, leading to racemization of the system. The balance between structures is easily shifting, e.g., isolysergic acid can be converted to lysergic acid by boiling in methanol or by barium hydroxide in high temperature. Moreover, the same effect can be seen in the natural racemic hydrazine derivatives of lysergic acid [35].

**Figure 8:**
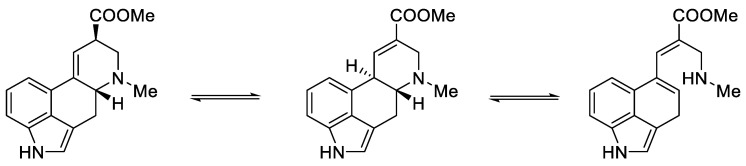
Equilibrium between the forms of lysergic acid, explaining the racemization mechanism.

Woodward, who was the first to perform a total synthesis of lysergic acid, proposed a reaction mechanism explaining epimerization at both chiral centers. These postulates were proved spectrally, experimentally, and crystallographically by Ramage two decades later. They proposed that the racemization process was via an achiral tricyclic intermediate (Figure 8), which could be formed by retro Michael fragmentation [36].

## 2. Methods of Lysergic Acid Synthesis

There are many methods of obtaining lysergic acid. They belong to two main types: using biotechnology and synthetic. In total, they allow for the production of 10–15 tons of acid annually [2].

The biotechnological method of obtaining lysergic acid is based on Arthur Stoll’s patent, who in 1918 proposed a method for the efficient separation and purification of ergotamine tartrate from fermented triticale and was put into production by Sandos in 1921. Then, pure lysergic acid can be obtained after amide hydrolysis [37,38].

The second method is proposed by chemists, and concerns the total synthesis of lysergic acid, starting with Kornfeld in 1956 [31]. To this day, 22 methods of synthesis of this compound have been described.

### 2.1. Woodward’s Strategy

#### 2.1.1. Woodward’s Research Group

**Scheme 1:**
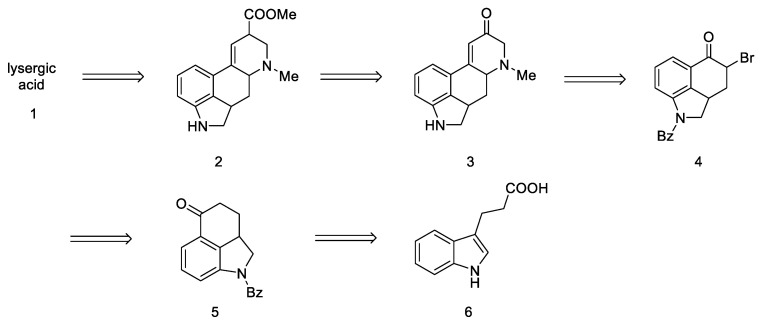
Retrosynthetic analysis leading to substrate in the Woodward strategy.

The first strategy for obtaining lysergic acid was proposed in 1956 by Woodward’s team [31]. The total synthesis assumed the preparation of the tricyclic ketone 5—1-benzoyl-2,2a,3,4-tetrahydrobenzo[cd]indol-5(1H)-one, then introducing the amine by adding the alkyl fragment at the C4 or C5 position and closing the D ring. The final step involved the isolation of the racemic product—lysergic acid **1** and confirmation of its structure by comparison to the authentic product (Scheme 1).

Since this is the first work on the preparation of lysergic acid, emphasis has been placed on presenting the line of reasoning described in this article. First, the synthesis of the tricyclic Uhle ketone **8** was presented, and then attempts were made to introduce substituents into it at the C4 and C5 positions. The next section describes attempts to functionalize the ketone by changing it into an unsaturated aldehyde. In the final section, the synthesis leading to the tetracyclic lysergic acid is shown.

**Figure 9:**
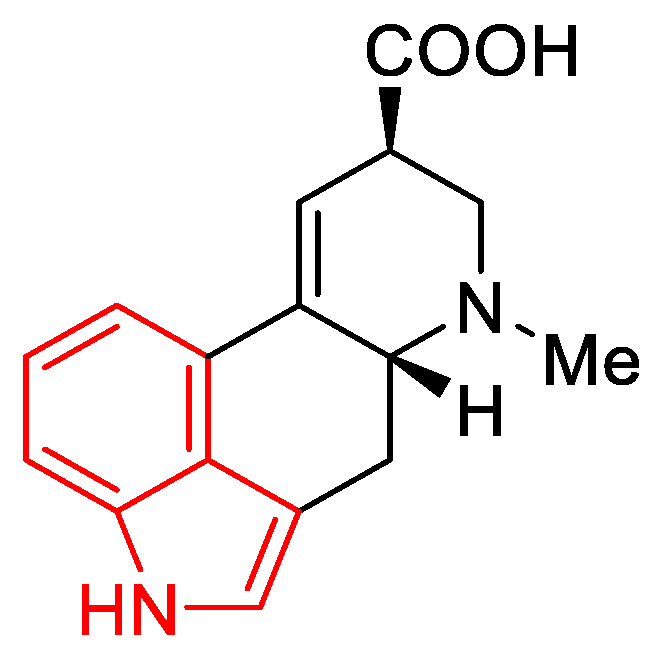
Indole scaffold (red) in the lysergic acid molecule.

**Preparation of the starting material.** There is a substituted indole system in the lysergic acid skeleton (Figure 9). Indoles are commercially available compounds, and their derivatives can be obtained in the laboratory according to many instructions in the literature.

The selection of the appropriate indole derivative was not so obvious, however, because the high reactivity of the hetero ring can produce undesirable byproducts based on a more stable structure of naphthalene. The greater stability of naphthalene derivatives is due to the higher resonance energy in relation to the indole [39].

**Scheme 2:**
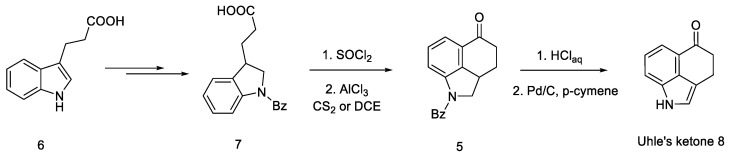
Obtaining Uhle’s ketone **8**.

The first step was to functionalize and reduce the starting material to the protected derivative of N-Benzoyl-3-(β-carboxyethyl)-dihydroindole **7** [40]. N-Benzoyl-3-(β-carboxyethyl)-dihydroindole was treated with thionyl chloride to the corresponding acid chloride and then converted into ketone **5** by the Friedel–Crafts reaction. Ketone **5** can be easily hydrolyzed with hydrochloric acid to the free base as well as dehydrogenated over carbon over palladium to the known 3,4-dihydro-benzo[cd]indol-5-one 8, known in the literature as the Uhle’s ketone (Scheme 2) [41].

**Figure 10:**
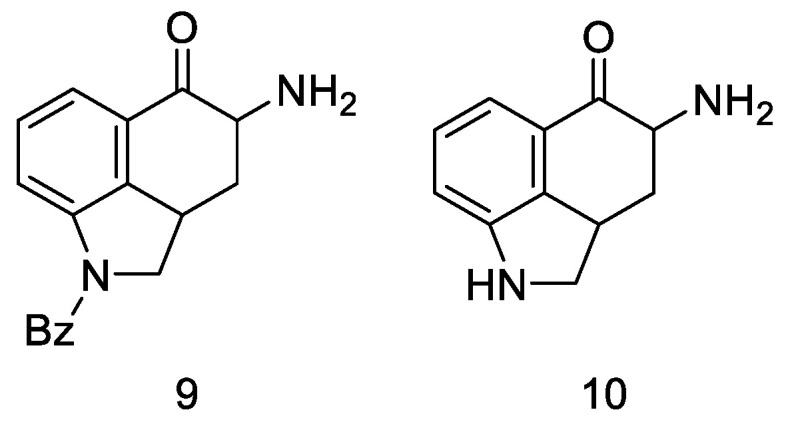
Structural formulas of amine derivatives (**9** and **10**).

**Very first attempts to obtain lysergic acid**. Using the obtained ketone **5**, attempts were made to introduce nitrogen into the C4 position (Figure 10).

**Scheme 3:**
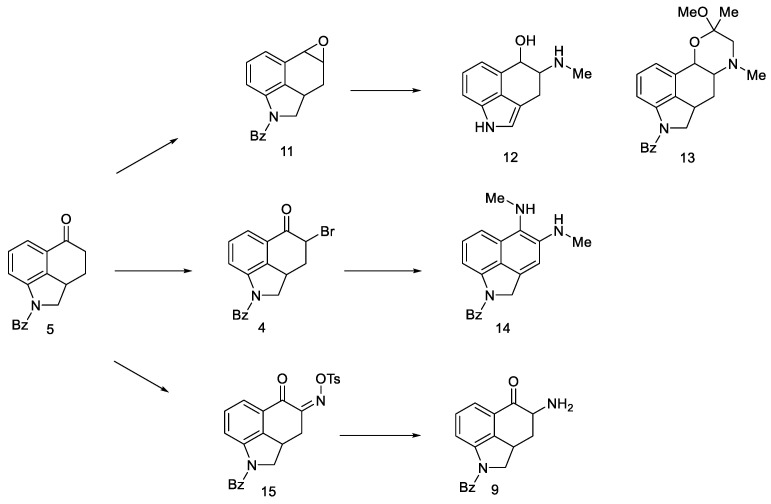
Derivative products strategies implemented.

Attempts were made to obtain compounds with free amine or diamine, but all attempts failed at various stages of the synthesis. The reaction of the 4-bromo derivative with methylamine had a complicated course, leading to the naphthalene derivative **14**. On the other hand, conversion of ketone **5** to O-toluenesulfonic oxime **15** allowed for obtaining the desired amine **9** (in the form of hydrochloride) by reaction with potassium ethoxide hydrochloride; however, when it was released from the salt, it was very unstable and immediately decomposed, just like diamine. The introduction of the CN and NHCHO functional groups into the C4 position was carried out with low yields; the reaction of oxirane derivatives with amines gave a number of ketal and hydroxyamine derivatives including **12**, **13** (Scheme 3).

**Scheme 4:**
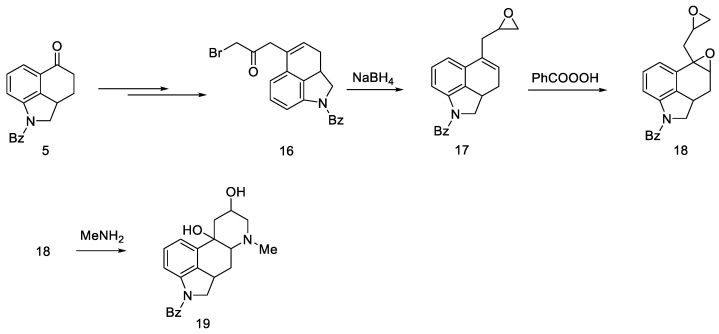
Diol **19** synthesis.

Adding a chain at the C5 position was explored as an alternative to introduction of nitrogen at the C4 position. For this purpose, the Reformatsky reaction was carried out with the use of ketone **5**. Treatment of bromoacetate in the presence of zinc gave a hydroxyester, which on heating in formic acid gave an unsaturated ester. The ester was converted to the bromoketone **16**, which was then reduced to the oxirane **17**. After using perbenzoic acid, dioxide **18** was obtained, which, after reacting with the base, gave tetracyclic diol **19** (Scheme 4).

The obtained product probably could be converted into lysergic acid, but the total yield of nine steps (from ketone **5** to tetracycle **19**) was so low that alternative paths were pursued.

**A successful synthetic route to lysergic acid.** This was achieved by synthesizing the unsaturated aldehyde **22** followed by the preparation of tertacyclic compounds.

**Scheme 5:**
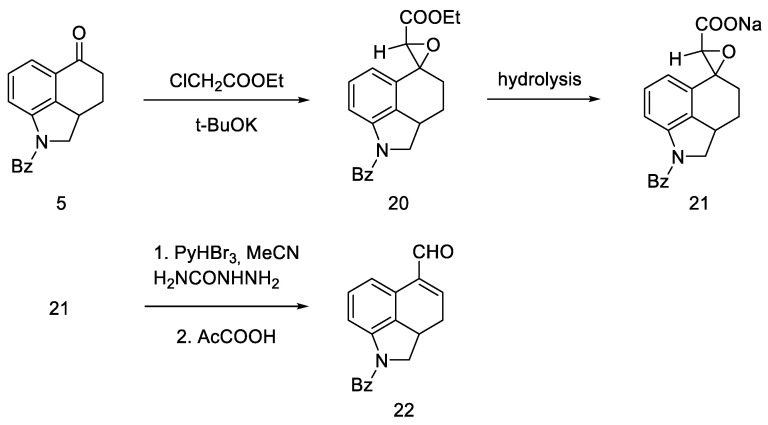
Aldehyde **22** synthesis.

This approach was to convert the ketone functional group in **5** into an unsaturated aldehyde **22.** To this end, a convergent synthesis leading to its preparation was developed using Darzens reaction [42], and then semicarbazide substitution followed by replacement with a pyruvic acid residue (Scheme 5).

**Scheme 6:**
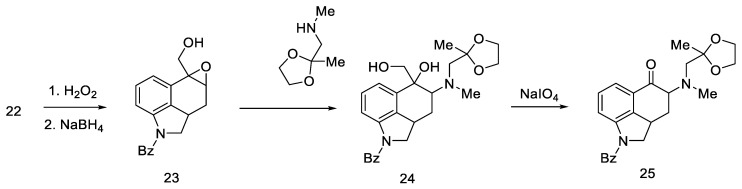
Ketone **25** synthesis.

Woodward proposed three approaches to synthesize an interesting intermediate. One of them turned out to be correct: the aldehyde **22** was converted to oxirane with hydrogen peroxide, then the -CHO group was reduced with NaBH_4_. Alcohol **23** was reacted with methylaminoacetone–ethylene ketal and oxidized, and the product—ketone **25** (Scheme 6)—was isolated with the desired substituted amino group at C4 position. The disadvantage of this approach is the low yield due to the ketal fragment attachment step.

**Scheme 7:**
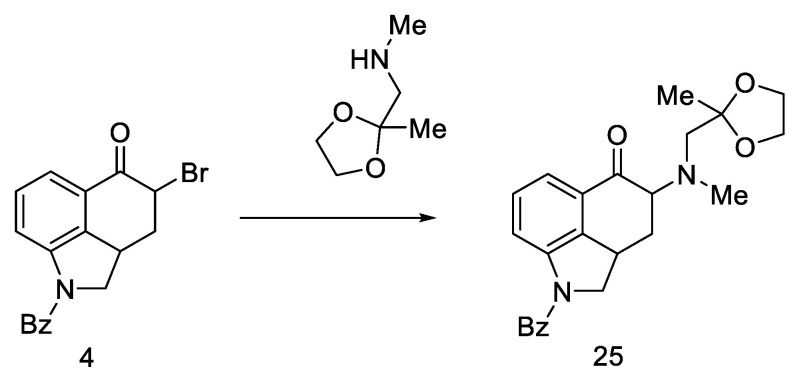
Reaction of the substitution of bromine **4** with ketal.

However, the reaction of the bromo substitution **4** with methylaminoacetone-ethylene ketal in a nonpolar solvent was tested, and product **25** (Scheme 7) was obtained in two steps from ketone **4** in high yield.

The last step involved closing the D-ring, substitution of the -COOH group, and dehydrogenation of the starting dihydroindole structure.

**Scheme 8:**
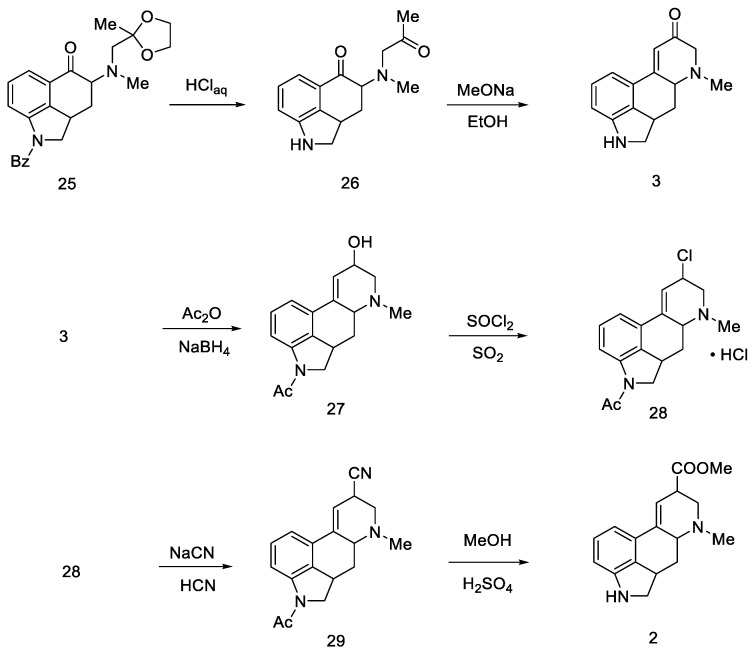
Woodward pathway to the deprotected key intermediate **2**.

First, protected compound **25** (Scheme 8) was hydrolyzed to a diketone, which was then cyclized. Then, α,β unsaturated carbonyl **30** was reduced with sodium borohydride to allyl alcohol **27**.

Optimization studies were performed according to Price and Krishnamurti, which described the conversion of allyl alcohol to allyl cyanide [43]. It had been found that good results can be achieved by forming chloride **28** followed by nitrile **29** using sodium cyanide in anhydrous liquid hydrogen cyanide. The -COOH group was displaced by methanolysis to an ester, followed by acidic or basic hydrolysis to compound **2**.

**Scheme 9:**
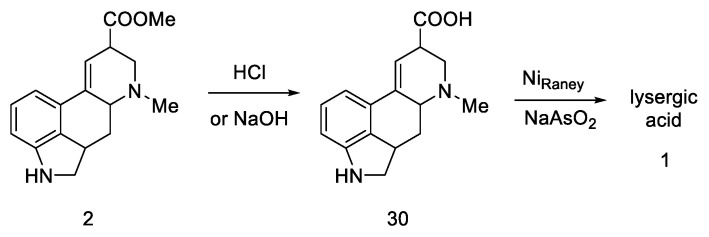
Woodward pathway to lysergic acid.

The final challenge was the oxidation of the B-ring, while maintaining the D-ring double-bond system. For this purpose, catalytic dehydrogenation on the Raney catalyst in the presence of sodium arsenate was used to obtain the target (±)-lysergic acid (Scheme 9) [44].

Parameters such as melting point, IR and UV spectra, X-ray powder diagrams, pKa, and chromatographic analyses were investigated, confirming the structure of the obtained target product [45,46].

In summary, Woodward’s strategy was based on starting from the indole dihydro derivative, obtaining a tricyclic ketone, introducing nitrogen at the C4 position via ketal, cyclization to the tetracyclic system, hydrolysis of the -CN group, and dehydrogenation of the lysergic acid derivative to the target acid. Final yield based on commercially available substrate was 0.8%.

**Figure 11:**
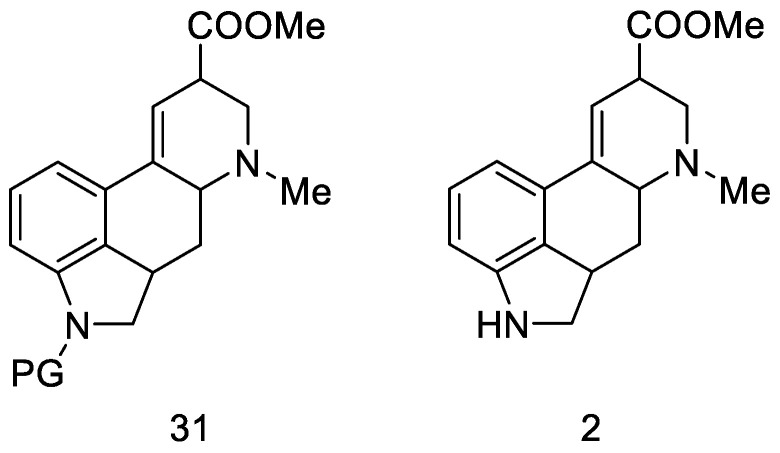
Woodward intermediate **31** and deprotected compound **2**.

The next part of this chapter presents syntheses using the key intermediate—hereinafter referred to as the Woodward intermediate (Figure 11), which is the ester **31** (PG—protecting group). It is puzzling that Woodward did not receive the product **31**. The name of this intermediate was given by researchers in the following years.

The synthesis step in Scheme 8 shows the simultaneous methanolysis of the nitrile group and deprotection of the amine, resulting in the product **2** being different from the intermediate **31** by a protecting group. However, the key intermediate can be converted into the product in a manner fully analogous to the procedure outlined by Woodward—lysergic acid **1** can be obtained from it by hydrolysis and dehydrogenation.

#### 2.1.2. Baillarge Group Research

**Scheme 10:**
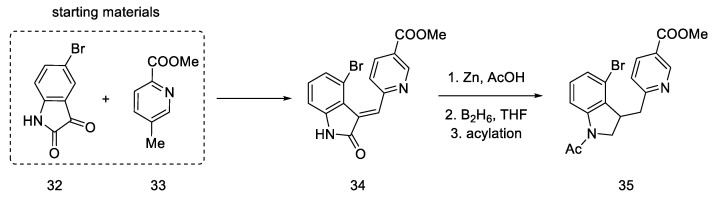
Beginning of the synthesis proposed by Baillarge team.

The lysergic acid synthesis performed by M. Julia on the Baillarge team [47] uses the preparation of a tricyclic system obtained as a result of the condensation of 5-bromoisatin **32** and methyl 6-methylnicotinate **33**. A key reaction was aryne cyclization. The synthesis was started by obtaining isatin nicotinate **34**. Thereafter, reduction of the double bond was performed by treatment with zinc in acetic acid as well as reduction with diborane to obtain a saturated indoline system. The amine was protected by acetylation to give product **35** (Scheme 10).

**Scheme 11:**
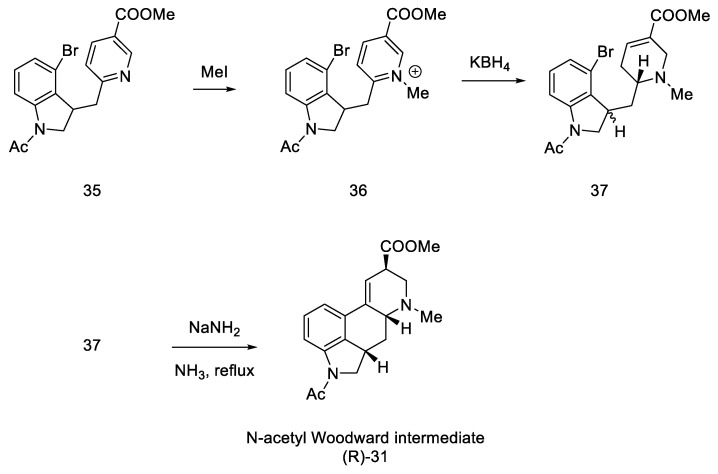
Synthesis of the protected Woodward’s intermediate.

In the next step, methyl iodide was added to compound **35**, followed by potassium borohydride, whereby the aromatic pyridine ring was reduced to α,β-unsaturated ester **37**. It was noted that a mixture of two diastereoisomers differing in physicochemical parameters was obtained. After the use of sodium amide, in ammonia in the reflux, the cyclization reaction was only observed for the (S)-isomer, with a yield of 36%. The product methyl N-acetyl-2,3-dihydrolysergate was isolated, which was in fact an Ac-protected Woodward intermediate **(R)-31** (Scheme 11).

Using the Woodward and Kornfeld procedure [31,32], this compound can be converted into lysergic acid **1**, which formally ends the synthesis. After examining the IR, UV, and mass spectra and preparing a TLC analysis in various systems, to compare the product with the intermediate obtained by Woodward, the identity of the obtained compound was confirmed.

#### 2.1.3. Ramage’s Research Group

R. Ramage, W. Armstrong, and S. Coulton, in 1981, used and improved Woodward’s strategy [32]. Optimization of the synthesis of tricyclic aldehyde **22**, which was then a substrate, in order to obtain Woodward’s intermediate **31**, was performed. A procedure was proposed, which consisted of passing through the Wittig reaction to the unsaturated diester **38** and then introducing the amino group using the Curtius degradation procedure and cyclization of the D ring.

In the work of the Ramage group, a lot of emphasis was placed on structures and intermediates through the analysis of NMR spectra, which confirmed the reaction mechanism proposed in the first synthesis. In addition to NMR studies, HPLC chromatogram was also investigated as were the crystallographic analyses and UV/IR spectra of the products. Thanks to these studies, it was found that lysergic acid formed as two epimers, and their ratio was determined.

**Scheme 12:**
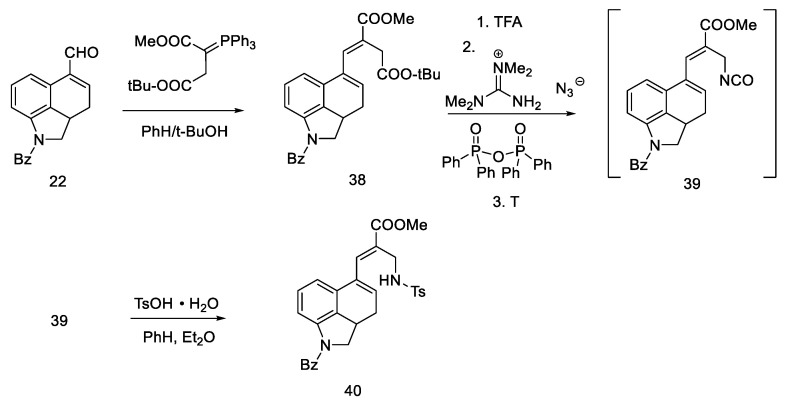
First part of the Ramage synthesis.

Aldehyde **22** (having a well-described, two-step synthetic pathway via semicarbazide in the experimental part) [32], was converted with a Wittig reaction through resonance-stabilized ylide into an unsaturated diester **38**. The reaction is stereospecific (configuration E), because the Wittig reaction with resonance stabilized ylides leads to systems having a carbonyl trans function to a larger group at the beta position [48]. Then, chemoselective hydrolysis of the ester bond of the -COOtBu group was carried out, and the Curtius degradation procedure was optimized (the total efficiency of this procedure was 80% [49]); at the beginning, tetramethylguanidine azide and mixed diphenylphosphinic anhydride were added to a diester **38**, and thermal rearrangement took place. Azide to the isocyanate **39** was performed, followed by hydration and decarboxylation to the amine. To avoid side reactions, it was decided to control the hydration using tosylic acid monohydrate. Thus, the amino group was introduced into compound **40** as a protected tosyl salt (Scheme 12).

**Scheme 13:**
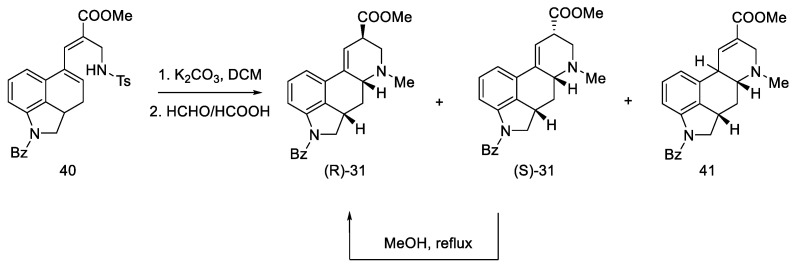
Second part of the Ramage synthesis.

Secondary amines have been shown to cyclize much more easily than primary amines [50]. The Woodward reaction mechanism model also assumes that the amine is an intermediate, spontaneously cyclizing to a 2,3-dihydrolysergic system [33,51]. Therefore, the Eschweiler–Clarke reaction [52,53] was performed with HCOOH/HCHO, yielding three tetracyclic products (**(R)-31**, **(S)-31**, **41**) in a 9:3:2 ratio, from which two Woodward intermediate epimers **(R/S)-31** were separated. The use of boiling methanol allowed for the epimerization of the isolysergic acid related compound **(S)-31** to the lysergic derivative **(R)-31** (Scheme 13).

**Scheme 14:**
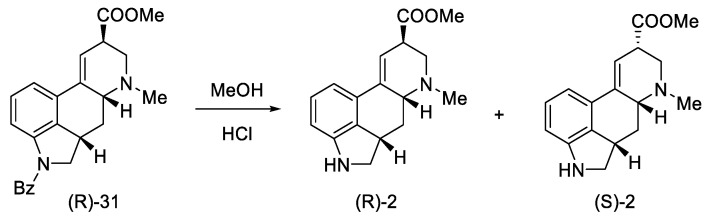
Obtained epimers of dihydrolysergic acid derivatives **2**.

Pure compound **(R)-31** was treated with HCl in MeOH to yield debenzylated epimeric lysergic acid derivatives **(R)-2** and **(S)-2** in a 5:2 ratio (Scheme 14). These products were identical to the material used in the first Woodward lysergic acid synthesis [31]. As a result of further work of the team, derivatives were also obtained, which were secondary and not tertiary amines in the D ring, enabling their further functionalization towards interesting derivatives.

Treating the Curtius procedure as a single one-pot reaction, the transition from ketone to Woodward intermediate consists of four reactions [54].

#### 2.1.4. Rebek’s Research Group

**Scheme 15:**
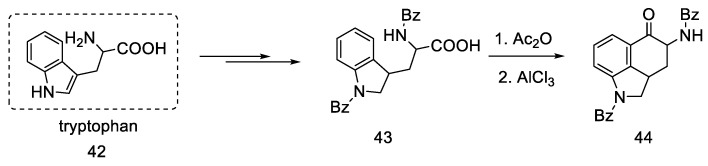
Preparation of the Rebek substrate **44**.

Rebek’s team proposed a different, simple starting material for the preparation of lysergic acid, which is the natural amino acid tryptophan **42** [55,56]. The first part involved obtaining the tricyclic ketone, analogous to the Woodward path [31]. Amino acid derivative **42** was converted to ketone **44** by adding acetic anhydride and aluminum chloride (Scheme 15).

The obtained ketone is functionalized by using the starting amino acid—it has an amino fragment in the C4 position.

**Scheme 16:**
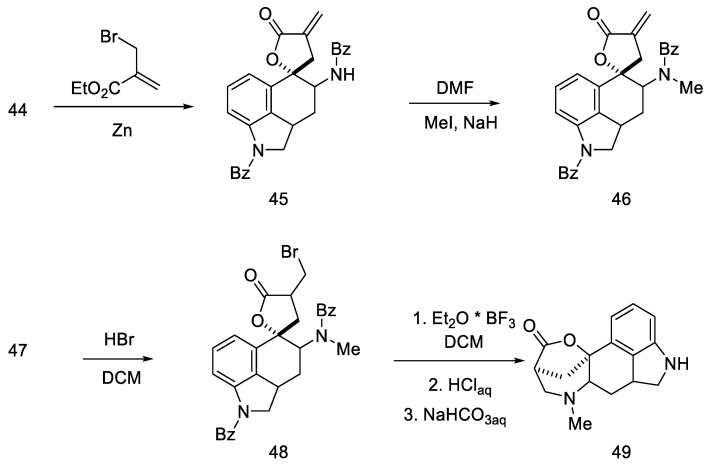
The first part of Rebek synthesis.

The next steps involved closing the D ring. Initially, ketone **44** was converted into spiro-lactone **45**, giving methylated compound **46** in the presence of sodium hydride and methyl iodide, which was hydrobrominated and then closed in the three-stage Henessian sequence [57] into the pentacyclic structure of the lactone **49** (Scheme 16).

**Scheme 17:**
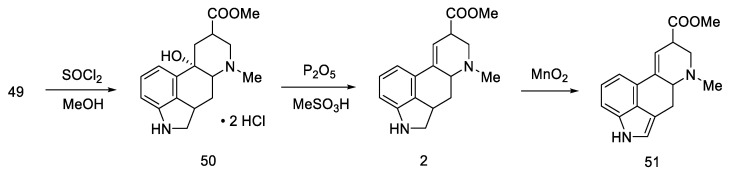
The second part of Rebek synthesis.

The last part was the formation of an ester group and dehydration to the lysergic acid structure. Lactone **49** was opened with thionyl chloride to dihydrochloride ester **50**, which was dehydrated to olefin **2**. Oxidation under mild conditions made it possible to obtain a mixture of deprotected isolysergic and lysergic acid esters **51** (Scheme 17), the physicochemical parameters of which were consistent with the data in the literature [58].

#### 2.1.5. Kiguchi’s Research Group

The Kiguchi group proposed, in 1982, the synthesis of the Woodward intermediate using the photochemical cyclization reaction of the D ring [59].

This work is an interesting attempt to obtain an optically pure intermediate. The original Woodward article [31] did not focus on obtaining pure chiral products. Instead, the lysergic acid that formed was a racemic mixture. The chirality of the intermediates was not tested either—as in Rebek’s work [55], a mixture of four final ester stereoisomers was obtained. Therefore, this procedure is linked to the Ramage article [32].

**Scheme 18:**
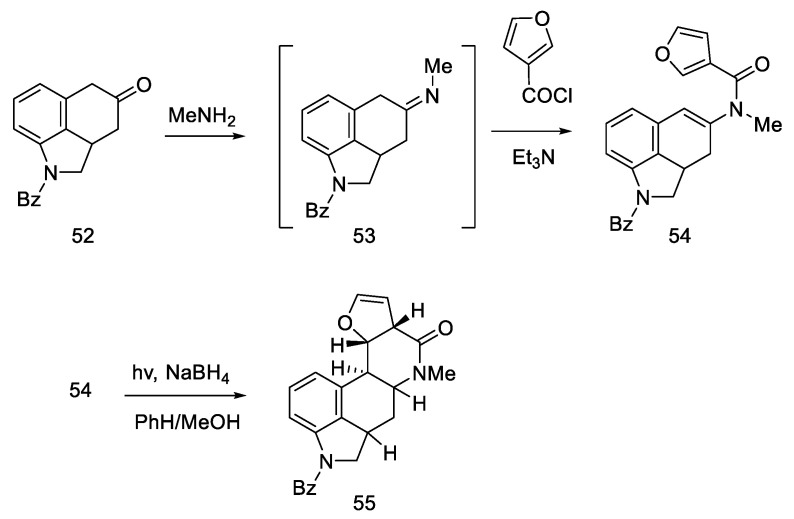
The first part of Kiguchi synthesis.

The synthesis started with a more complicated structure than proposed by Woodward or Rebek—with the tricyclic ketone (benzo(f)quinoline derivative) described in the literature **52** [60,61]. Known ketone **52** was treated with methylamine to give imine **53** as an intermediate, which was easily converted to enamide **54** after acylation with 3-furoyl chloride in the presence of triethylamine. Reductive photocyclization was performed, leading to lactam **55** (Scheme 18).

**Scheme 19:**
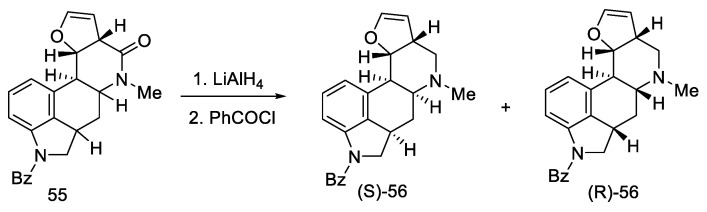
The second part of Kiguchi synthesis.

Reduction of lactam **55** with LiAlH_4_ followed by reprotection of the amine gave a mixture of two diastereoisomers **56** (Scheme 19). The decarbonylated amine **(R)-56**, having the stereochemistry corresponding to the *d*-(+)-configuration of lysergic acid, was isolated by crystallization.

**Scheme 20:**
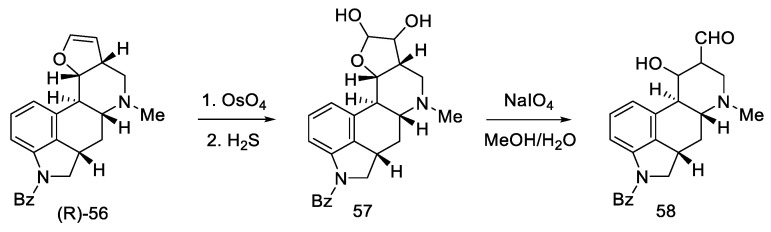
The third part of Kiguchi synthesis.

Oxidation of amine **(R)-56** gave glycol **57**, which is a diastereomeric mixture of two *cis*-glycols. Without further purification, oxidation of the glycol with sodium periodate gave a mixture of epimeric hydroxyaldehydes **58** (Scheme 20) [59].

**Scheme 21:**
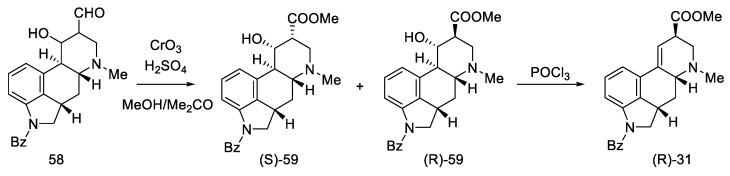
The last part of Kiguchi synthesis.

After oxidation of hydroxyaldehydes to hydroxyesters, it was investigated that the products contain two epimers, which are precursors of isolysergic **(S)-59** and lysergic **(R)-59** acids. Dehydration of both hydroxyesters gave one product—unsaturated ester **(R)-31**, which is an optically pure Woodward intermediate (Scheme 21). This selectivity is due to the higher thermodynamic stability of β-equatorial esters relative to the α-axial esters [33,51]. Spectral examination confirmed the identity of the structure to the compound obtained by Ramage [32] and Woodward [31].

**Scheme 22:**
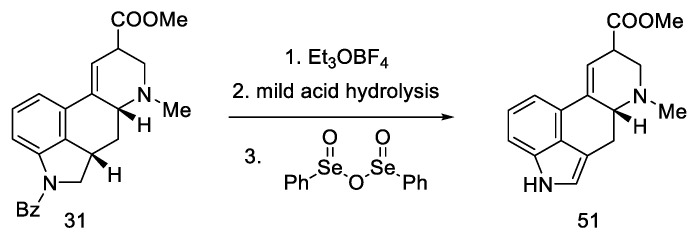
Additional Kiguchi research.

Continuing the work of the team, during the first synthesis of (±)-isofumigaclavine B in 1985 [62], the synthesis of lysergic acid was almost completed. Removal of Bz protecting group and oxidation of mixed epimers of ester (**31**) produced lysergic acid ester **51** (Scheme 22).

The use of the photochemical cyclization reaction is an interesting alternative to the previous proposals for the closing of the D-ring. It is a completely fresh approach, using a different branch of chemical synthesis. This work was an important step towards obtaining pure chiral *d*-lysergic acid.

**Scheme 23:**
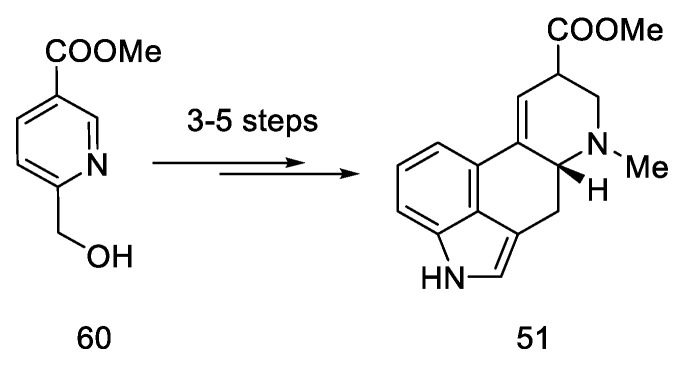
A new method for the synthesis of compound **51**.

According to the latest reports, structure **51** can also be obtained in three–five steps from a nicotinic acid ester **60** (Scheme 23) [63].

### 2.2. Hendrickson’s Strategy

#### 2.2.1. Direct Hendrickson’s Research

Hendrickson’s group approached the topic of lysergic acid synthesis from a new angle. Using readily available components such as an indole halide and a pyridine derivative, they obtained the lysergic acid structure using a coupling reaction followed by cyclization of the aldehyde to give the D ring—the key Hendrickson intermediate [64]. The desired product was then obtained by reduction with a classical borohydride. The main advantage of this approach is the convergence and simplicity of the method, as well as the total efficiency. The product was obtained in eight steps, and the yield was 10.6%.

The assumptions of the strategy proposed by Hendrickson are: synthesis of lysergic acid through the shortest possible path, without protection of the indole fragment, and introduction of the D ring through Suzuki coupling.

**Scheme 24:**
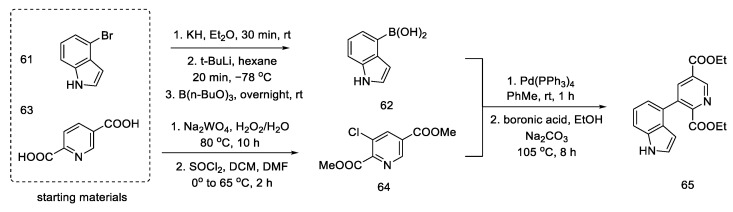
Coupling reaction leading to a tricyclic system **65**.

The synthesis was started with the preparation of the halogen derivative of indole **61**, which was converted into compound **62**. Starting from the dicarboxylic derivative of pyridine **63**, the synthesis of its N-oxide was performed, and then, with thionyl chloride, it was converted into a chlorine derivative of **64** (interestingly, only under these conditions the meta derivative is obtained, instead of the classic ortho/para substitution). The aromatic substrates, thus prepared, were easily coupled via a palladium-catalyzed Suzuki reaction to the tricyclic derivative **65** (Scheme 24). An analogous coupling was simultaneously described by Doll [65].

**Scheme 25:**
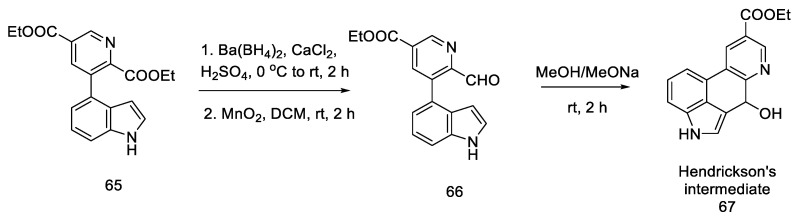
Obtaining Hendickson’s intermediate.

Closing the tetracyclic system posed some difficulties—diester **65** did not give the expected cyclization product; therefore, the ester was reduced to alcohol and then gently oxidized to active aldehyde **66**, which, under basic conditions, resulted in a tertiary cyclic alcohol **67**, known as Hendrickson’s intermediate (Scheme 25).

**Scheme 26:**
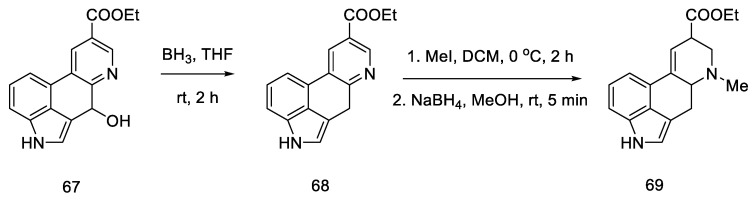
Synthesis of lysergic acid ester proposed by Hendrickson.

Reduction of the alcohol **67**, N-methylation of the pyridine derivative and reduction of the aromatic system were performed to obtain a mixture of **69**: lysergic acid ester and isolysergic acid ester in a ratio of 6:1 (Scheme 26). The last three steps were performed without isolation to avoid losses due to product instability. Using the procedure from the literature on the isomerization of the iso-lysergic acid form, it was converted to the desired acid under basic conditions. Its physicochemical properties such as melting point were checked to confirm its structure.

However, this pathway of obtaining lysergic acid was proven to be not feasible, as described later in this chapter.

#### 2.2.2. Problems Noticed by Bekkam

In 2011, Bekkam, Mo, and Nichols [66] repeated the synthesis of lysergic acid using the conditions proposed by Hendrickson [64], using methyl esters **70**. Surprisingly, it turned out that the cyclization step to compound **67**, as shown in Scheme 25, did not occur.

**Scheme 27:**
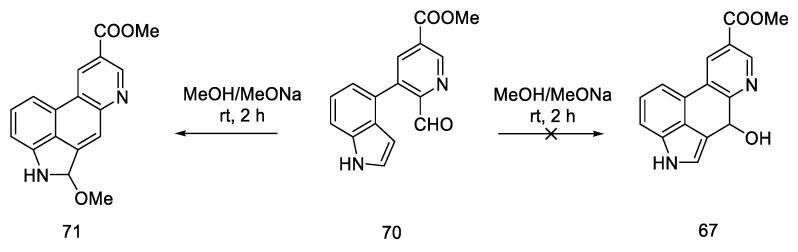
Structural formula of the product obtained in the literature procedure repeated by Bekkam.

The reaction was attempted multiple times; neither the change of researcher nor the multiple repetitions of the reactions with different amounts of base, different time, and different temperatures resulted in the desired effect. The obtained compound **71** was the product of the cyclization and aromatization of the system (Scheme 27).

The structure was unambiguously checked and determined by melting point and the analysis of one- and two-dimensional NMR spectra (^1^H, ^13^C, COSY, HMBC, TCOSY, HMQC).

The reason for obtaining the other main product is the resonance stabilization of the indoquinoline system, as well as the fact that the dihedral angle between the carbonyl carbon and the C3 carbon is 160°, which allows for easy elimination under basic conditions according to the E2 mechanism.

Researchers have not been able to contact members of Hendrickson’s group, but it must be assumed that this particular path is not feasible. 

However, before these problems were noticed by Bekkam, Hendrickson’s strategy was used to formally synthesize lysergic acid by Yigang Zhao [67] and Beaundry [68].

#### 2.2.3. Yigang Zhao Work

Yigang Zhao’s doctoral dissertation from the Snieckus group presents an alternative method of obtaining the key Hendrickson intermediate [67,69]. Selective amidation, DoM (Directed Orto Metalation-Suzuki cross coupling) cross coupling, and the chemoselective reduction of an amide to an aldehyde using Schwartz’s reagent were used for this purpose. This simplifies Hendrickson’s strategy by eliminating one step. Then, according to the reactions in the literature, the compound can be deprotected and cyclized [64].

**Scheme 28:**
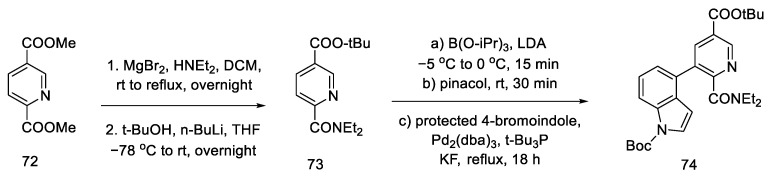
Coupling reaction leading to a protected tricyclic system **74**.

The first step was selective amidation and transesterification to **73**. The substrate prepared in this way can be coupled using the conditions optimized by the researchers for a tricyclic system **74** (Scheme 28). The reduction step with the use of an organozirconium reagent took place after protection of the indole N-H; therefore, Boc-protection was proposed.

**Scheme 29:**
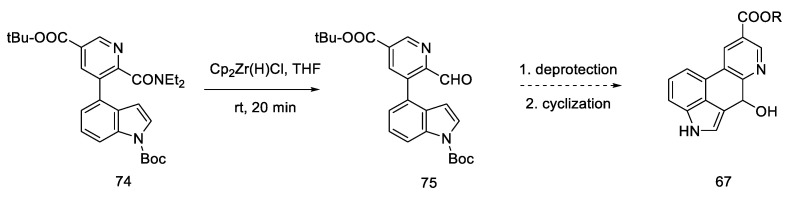
Synthetic path leading to the Henrickson’s intermediate, as postulated by Yigang.

Carrying out the reduction with the Schwartz reagent allows for obtaining the aldehyde in an efficient manner, even in the presence of a more reducible ester. It was postulated (dotted arrow) that in this way, after carrying out two steps, it could lead to the Hendrickson’s intermediate **67** (Scheme 29).

This modification makes it possible to obtain the intermediate in six, instead of seven steps, but it involves the use of organometallic reagents such as BuLi and Cp_2_Zr(H)Cl.

#### 2.2.4. Beaundry’s Research

During their research, Beaundry’s group proposed the formal synthesis of lysergic acid via the Hendrickson intermediate as an example of using this method for the preparation of substituted indoles [68]. The synthesis relies on the coupling of a terminal alkyne alcohol and a halogen derivative of the pyridine ester **76** and the substitution of an amine with a cyclic substituent, followed by the closure of the indole system, leading to the known precursor **65**, from which the Hendrickson’s intermediate can be obtained in two steps.

**Scheme 30:**
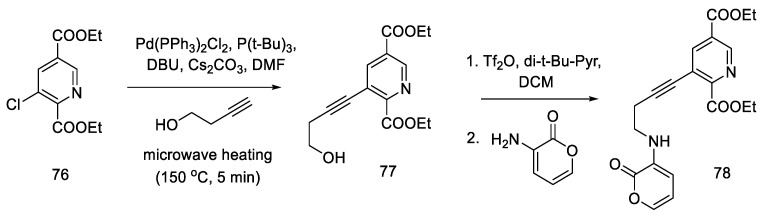
Beginning of the synthesis proposed by Beaundry.

Chloropyridine **76** was prepared according to the procedure in the literature [64]. The Sonogashira coupling of the alkyne and chloropyridine was performed using microwave heating. Compound **77** was then activated with triflate anhydride and converted to a secondary amine **78** (Scheme 30).

The research of the Beaundry group focused on the preparation of the bicyclic system of substituted indole, and, as a result, the methods of oxidative cyclization were optimized: in the first step, butyl nitrile was added under basic conditions and strongly heated with microwave, and then HO-TEMPO was added in the presence of copper (I) salt and controlled amounts of oxygen to give the indolyl-pyridine **65** in good yield.

**Scheme 31:**
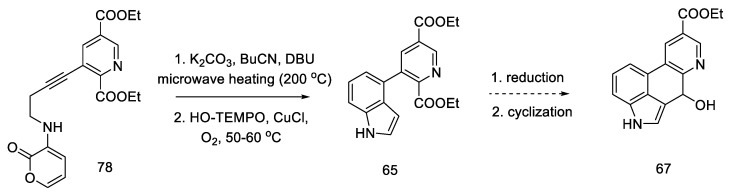
Ending of the synthesis proposed by Beaundry.

Hendrickson’s intermediate **67** can be obtained in two steps, by the reduction and cyclization described above (Scheme 31) [64].

### 2.3. Szantay’s Strategy

#### 2.3.1. Direct Szantay’s Research

Szantay proposed the synthesis of lysergic acid using an aldol condensation reaction to close the D ring. The first presentation of the synthesis of (+)-lysergic acid is also a great achievement, thanks to the use of the obtained tetracyclic system, which was separated by crystallization with a chiral auxiliary. In doing so, he obtained a key intermediate **(R)-82** (Szantay’s intermediate). The next four steps are required to obtain lysergic acid. The article also describes, in addition to the lysergic acid total synthesis, the synthesis of an important derivative of ergocriptinine, which is a peptide derivative of lysergic acid on a large scale [70].

The synthesis involved going through the following steps: obtaining a tricyclic ketone, closing the fourth ring using the aldol reaction, enantioselective separation of the obtained product, and substituting the carbonyl group with a carboxylic acid to obtain (+)-lysergic acid.

**Scheme 32:**
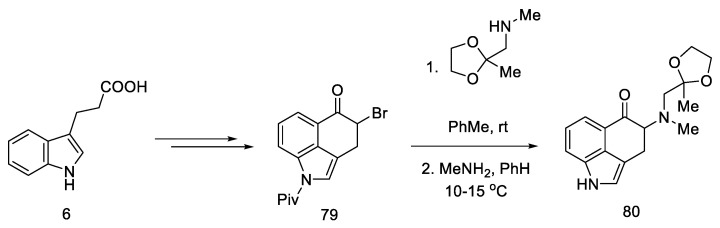
Beginning of the synthesis proposed by Szantay.

The synthesis was started with the preparation of the bromo derivative of Uhle’s ketone **79**. The original procedure for the preparation of Uhle’s ketone assumed starting from the 3-indolpropionic acid **6** by the Goto method [71]; however, only the preparation of the bromo derivative made it possible to carry out the amination reaction. The authors proposed two methods of obtaining compound **80** from the bromo derivative **79** (Scheme 32 shows a two-step synthesis with a yield of 35%, but an alternative is a four-step process with a total yield that is higher by 4%). Next, the NH group of the indole was deprotected.

The next step involved closing the D ring. For this purpose, intramolecular aldol condensation was employed, using 1,5-dicarbonyl moiety **81,** formed after the deprotection of ketone **80**. However, selecting the optimal conditions was a difficult task. Bases ranging from tert-butoxide to bases such as LiHMDS were tested with no effect. A satisfactory effect was achieved using the Eschenmoser conditions—LiBr and triethylamine [72].

The authors suggest that the tetracyclic product **82** is formed by lithium bromide activation of the carbonyl groups in the presence of an amine, due to the higher affinity of lithium for oxygen than nitrogen. The presence of the amine is essential—it is used to abstract a proton from the alpha position next to the carbonyl group. These initial synthesis steps make use of the synthesis intermediates already proposed by Woodward [31].

**Scheme 33:**
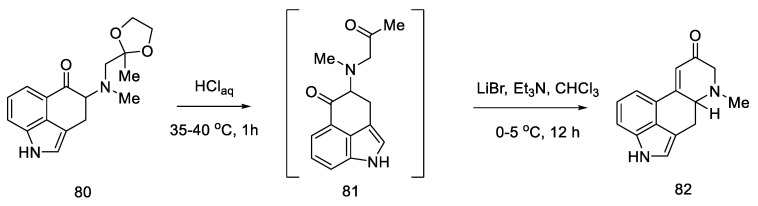
Preparation of the carbonyl α,β-unsaturated system **82**.

The best effect was achieved by performing the deprotection and cyclization reaction without isolating compound **81** and isolating compound **82** by crystallization to give a total 60% yield of these steps (Scheme 33).

**Scheme 34:**
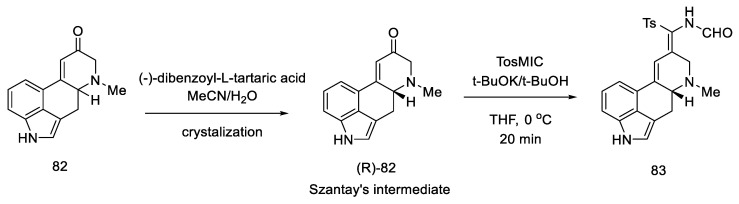
Obtaining Szantay’s intermediate.

Enantioselective separation was performed by crystallization with chiral dibenzoyltartaric acid, and pure compound **(R)-82** was obtained, contributing to the first asymmetric synthesis of (+)-lysergic acid. The physicochemical properties of the obtained compound were compared with the degradation product of natural (+)-lysergic acid. The optically pure isomer **(R)-82**—Szantay’s intermediate—was reacted with isonitrile to produce a tosylated formamide derivative **83** (Scheme 34).

**Scheme 35:**
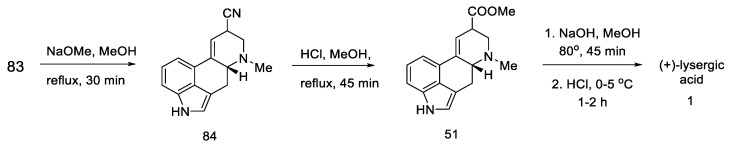
Synthesis of lysergic acid proposed by Szantay.

Basic hydrolysis gave the R/S nitrile mixture (**84**), which was converted to the R/S methyl ester mixture **51** by Pinner reaction. Basic ester hydrolysis gave pure (+)-lysergic acid by epimerization (Scheme 35) [70].

#### 2.3.2. Continuation of Szantay’s Research

**Figure 12:**
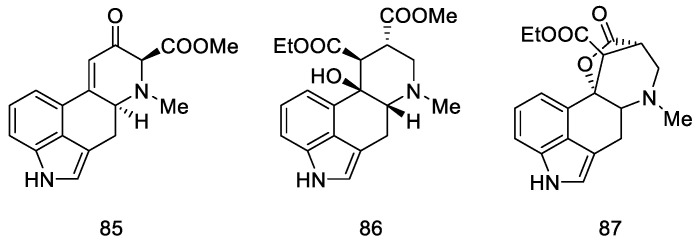
Structural formulas of compounds obtained in further works of Szantay’s research group.

A year later, in 2005, *Helvetica Chimica Acta* published a review presenting the entire range of works by Szantay’s group [73]. Unfortunately, the only successful attempt to obtain lysergic acid was the synthesis presented in Section 2.3.1, although the work resulted in a number of interesting four- and five-cyclic compounds based on the ergoline structure, obtained by Stobbe (Figure 12).

#### 2.3.3. Garner’s Research

In 2021, Garner and Rathnayake published a study that used Szantay’s intermediate to obtain lysergic acid [74]. This is a novel approach that involves carrying out an intramolecular cycloaddition reaction of an azomethine ylide, generated in situ, followed by ring expansion to Szantay’s ketone. During these reactions, C and D’ rings (where D’ is a five-membered precursor of the D ring in the ergoline structure) are obtained, and then they are transformed into CD rings. The disadvantage of this method is a very large number of stages—Szantay’s intermediate **(R)-82** is obtained in 16 stages, with a total yield of 1.0% (average—75% per step).

**Scheme 36:**
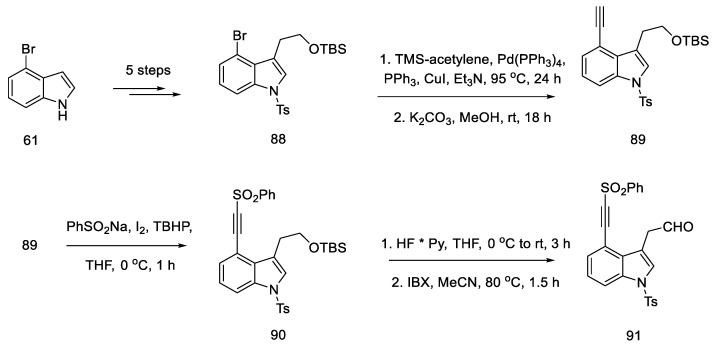
Beginning of the synthesis proposed by Garner.

The synthesis was started with 4-bromoindole **61**, which was converted into the bromoindole **88** derivative, according to a procedure in the literature [75]. Then, using a Sonogashira coupling and removal of the TMS group, terminal acetylene **89** was obtained, which was substituted with the sulfonyl group. The obtained compound **90** was deprotected to the form of an alcohol and oxidized to aldehyde **91**, which was a substrate for the intramolecular cycloaddition reaction (Scheme 36).

**Scheme 37:**
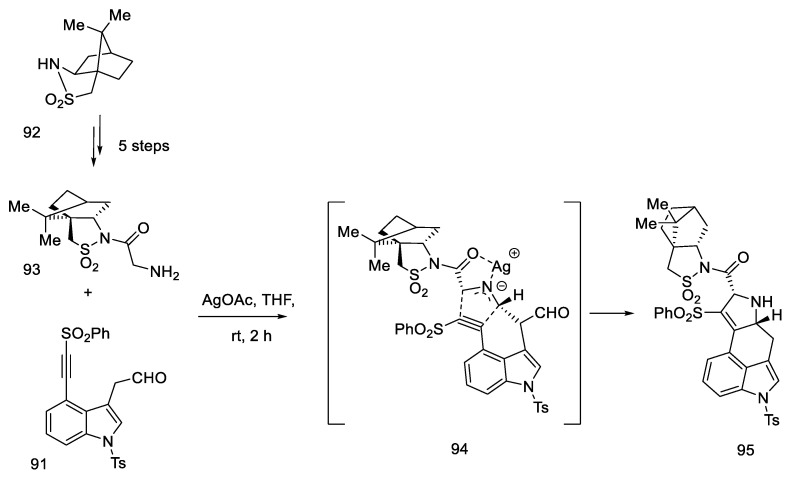
Preparation of a tetracyclic derivative **95** with a camphor substituent.

The second substrate was compound **93**, a chiral glycylsultam, obtained in a five-step synthesis from (2S)-bornane-2,10-sultam **92**, a camphor derivative. The steps of substituting the acid halide with an organolithium agent were performed, followed by the introduction of the -NH2 group by the azide. The intramolecular cycloaddition of an azomethine ylide is an asymmetric cycloaddition reaction of three elements: aldehyde, dipolarophilic factor (in this case in one substrate **91**), and glycylsultane. As a result of the reaction—as the authors suggest—through the intermediate **94**, a system of four ABCD rings is formed (Scheme 37) in an enantioselective reaction.

In the following steps, a fragment of the camphor derivative was removed, and the D ring substituent was reduced, yielding alcohol **97**. Then, using the five- to six-membered ring expansion reaction proposed by Cossy and Charette [76,77], the correct system was obtained **98**.

**Scheme 38:**
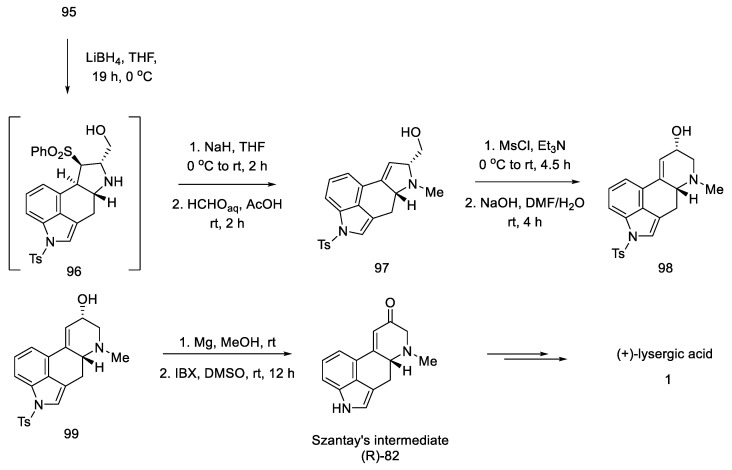
Synthesis of lysergic acid proposed by Garner.

The oxidation and deprotection of compound **99** allowed for obtaining Szantay’s intermediate **(R)-82**, from which the proper, optically pure (+)-lysergic acid can be obtained in four steps (Scheme 38).

### 2.4. Closing the C/D-Ring Using the Heck Method

The Mizoroki–Heck reaction, including the intramolecular version, is the reaction of the selective coupling of an aryl or vinyl halide and an alkene in the presence of a palladium catalyst under basic conditions [78]. It is often used to enclose the six-membered C-ring in the ergoline structure. A lot of synthetic work was based on the scheme: preparation of halogen-indole or tryptophan derivatives, then coupling of the D-ring, manipulation of substituents to create favorable conditions for the intramolecular Heck reaction, and conversion of the functional groups to (+)-lysergic acid.

#### 2.4.1. Ortar and Kurihara Groups

**Strategy of closing D-ring using the Heck method**. In 1988, the Ortar group was the first to propose the preparation of lysergic acid through the palladium-catalyzed Heck coupling [79]. The starting material in the synthesis was Uhle’s ketone derivative **5** [31].

The tricyclic olefin **100** was obtained, as a substrate for the coupling reaction, after the use of a pyridine derivative and triflic anhydride. The second substrate was the unsaturated amino ester **102**, which was obtained by carbanion generation from amino ester **101,** the addition of formaldehyde, the introduction of mesyl chloride, and elimination promoted by strong base DBU.

**Scheme 39:**
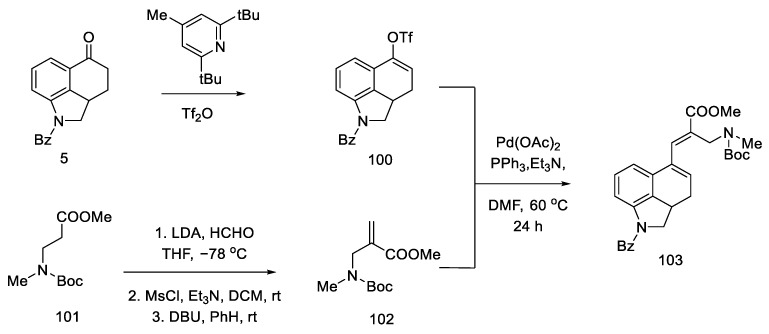
Preparation of a tricyclic system by Heck coupling as the source of the D-ring.

Heck coupling resulted in compound **103**, which readily cyclizes in the presence of hydrochloric acid (Scheme 39).

**Scheme 40:**
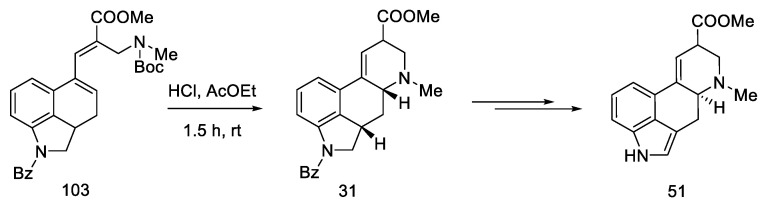
Synthesis of lysergic acid ester **51** proposed by Ortar.

A mixture of the two epimers **31** was obtained with a yield of 60%, in a ratio of 1.7:1. Despite being consistent with the spectral data reported by Ninomiya and Rebek, refluxing the ester mixture **31** in methanol did not result in a 3:1 ratio, as reported in the literature [54,58]. Nevertheless, using the obtained mixture of esters, the target product can be prepared, which ends the formal synthesis of lysergic acid (Scheme 40) [31,32].

**Kurihara group research.** Two years earlier, the Kurihara group proposed a method of obtaining lysergic acid using the same substrates as Ortar, reaching the same analogous intermediate, which can be cyclized; however, in this case, a ring-closing reaction was performed without using the Heck coupling method [80,81]. The same intermediate that was already described by Woodward is obtained [31].

The substrate for the proposed synthesis was the Uhle ketone derivative **5** [31]. Converting the carbonyl functional group first to nitrile and then to aldehyde via DIBAL-H reduction gave compound **22**. Then, using a strong organolithium base, LDA, introduction was performed into the tricyclic structure of the amine-ester moiety.

**Scheme 41:**
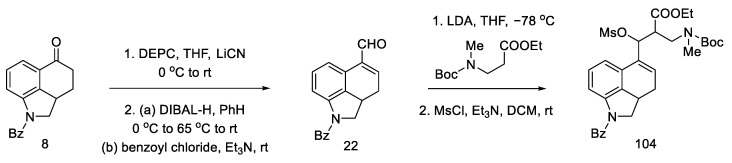
Preparation of a tricyclic system by Kurihara, analogous to that of Ortar.

In the next stage, the alcohol was changed to the -OMs group, obtaining the mesylate **104** (Scheme 41).

**Scheme 42:**
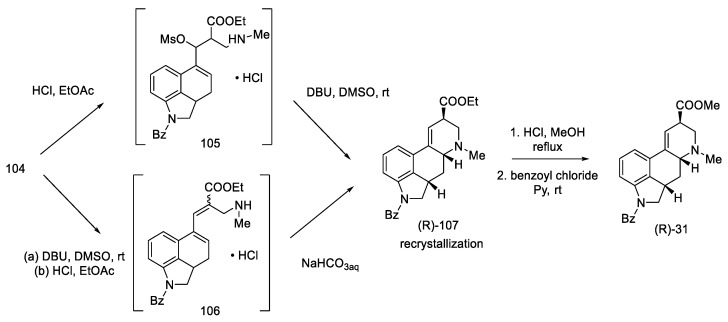
Preparation of a lysergic acid protected ester **(R)-31** by the Kurihara strategy.

Conversion into Woodward’s key intermediate ethyl ester could be carried out in two ways: either **104** was deprotected first and then cyclized, or it was first treated with DBU, and, in the next step, the protecting group was removed. The reaction runs through intermediate products, which was used by the Ortar group [79]. Three products were obtained: a mixture of (R) and (S) of the expected N-benzoy1-2,3-dihydrolysergate and also the double-bond isomerization product. Recrystallization of the mixture in ethyl acetate allowed for the isolation of the pure product. The last step was hydrolysis and re-esterification, leading to the known compound **(R)-31**, from which, according to the literature [62], lysergic acid can be obtained (Scheme 42).

#### 2.4.2. Fukuyama’s Research

**Strategy I**. In 2009, the Fukuyama group proposed the synthesis of (+)-lysergic acid [80], which so far had only been possible through the Szantay approach [73]. Lysergic acid was prepared by closing the C-ring via an intramolecular Heck reaction using the tricyclic compound **117**. In turn, this compound could be obtained from the bromomagnesium indole derivative and from the disubstituted tetrahydropyridine. Chiral tetrahydropyridine, the structure of which corresponds to the D-ring, was obtained from L-pyroglutamic acid by a dissrotational electrocyclic reaction [82].

**Scheme 43:**
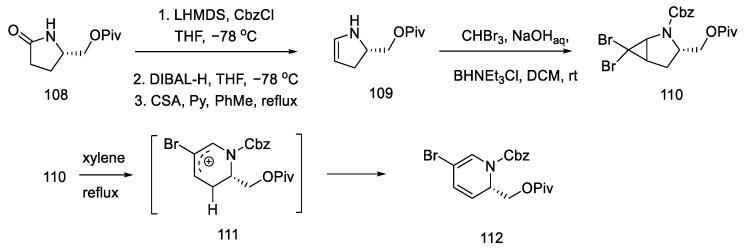
Synthesis of dihydropyridine **112**.

The synthesis began with the acylation of lactam **108** and the reduction and dehydration of the hemiaminal to enamide **109**. Using catalysis and two-phase conditions, alkene was converted to dibromocyclopropane **110,** which rearranged at high temperature into carbocation **111** and then into the substituted dihydropyridine **112** (Scheme 43).

**Scheme 44:**
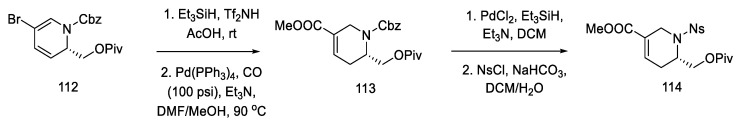
Protection of the tetrahydropyridine with a nosyl group.

As a result of the next seven stages of the synthesis, a tetrahydropyridine acid chloride **115** was prepared, which could be subsequently coupled to a tricyclic system using an organometallic compound. The resulting compound **112** was reduced and then carbonylated under catalytic conditions. Using a Ritter’s method [83], the Cbz protecting group was replaced with a -Ns group (Scheme 44). Then, it was possible to apply the Jones oxidation with chromium oxide, and the compound obtained in this way was transformed into acid chloride **115** by the Ghosez procedure [84]. Interestingly, this is the first example of the synthesis of optically pure dihydropyridine from L-pyroglutamic acid. As a result, a chiral center is introduced into the compound, which makes it possible to obtain pure (+)-lysergic acid.

**Scheme 45:**
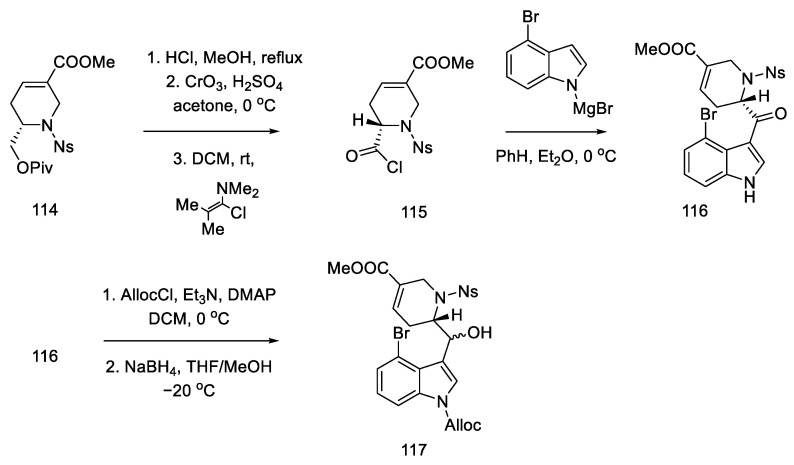
Synthesis of tricyclic alcohol **117**.

The coupling of the D-ring derived from piperidine **115** and the 4-bromoindole derivative was performed in moderate yield. The nitrogen in the indole was protected with Alloc, whereby the carbonyl group was activated and reduced to a diastereomeric mixture of alcohol **117** (Scheme 45).

**Scheme 46:**
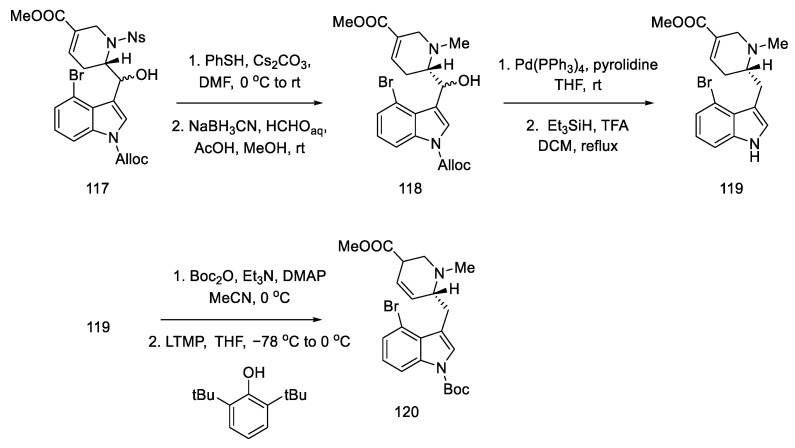
Preparation of ester **120**.

Subsequent reactions involved the deprotection and methylation of tetrahydropyridine nitrogen to give alcohol **118**. Next, indole nitrogen was deprotected, and the alcohol was reduced with triethylsilane under acidic conditions. The indole nitrogen was -Boc protected, and the double bond was isomerized with 2,6-tert-butylphenol and lithium tetramethylpiperide to give a mixture of diastereomeric esters **120** (Scheme 46).

**Scheme 47:**
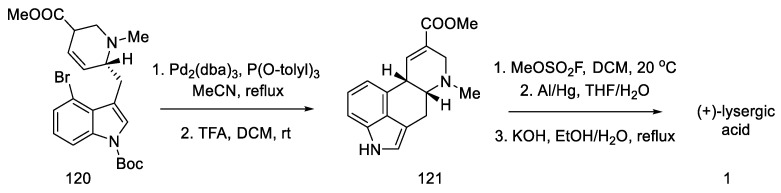
Preparation of lysergic acid in Fukuyama strategy I.

These isomers were separated and subjected to an intramolecular Heck reaction closing the C-ring of the ergoline structure to obtain the protected ester. The desired trans isomer was obtained in excess (3:1). After deprotection of the indole nitrogen with trifluoroacetic acid, ester **121** was obtained, which can be converted to lysergic acid (Scheme 47) [85].

Despite the undoubted advantage of the approach proposed by Fukuyama, which allows for obtaining pure (+)-lysergic acid, there are many disadvantages associated with it. First of all, the number of stages, since the procedure involves over 30 steps from the starting lactam to the final product. Additionally, the use of a large number of protecting groups should be mentioned as a consequence of the use of numerous organometallic compounds, such as DIBAL-H, LTMP, or Grignard compounds, and other requirements related to such active reagents, for example temperature control. Realizing the disadvantages of the proposed approach, Fukuyama introduced numerous alterations in subsequent publications.

**Strategy II.** The Fukuyama group continued to work on new methods of obtaining lysergic acid. The aim was to simplify and shorten the synthesis and increase the overall yield. A synthesis based on a one-pot double-cyclization reaction, simultaneously closing the B and C rings, thanks to an intramolecular amination reaction to the aromatic system (to the indole AB system) and the palladium-catalyzed Heck reaction (ring C), have been proposed [86]. In order to obtain the substrate for the double-cyclization reaction, the synthesis of compound **131** was carried out by the coupling method of dibromo-iodobenzene and nitrated olefin **127.** The synthesis uses 2-(but-3-en-1-yl)-propane-1,3-diole **122** as a starting material.

**Scheme 48:**
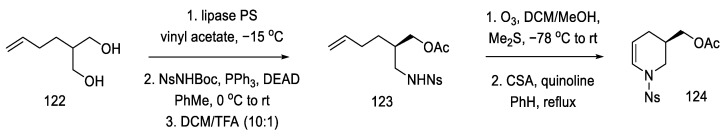
Synthesis of cyclic acetate-enamine **124**.

The synthesis of the optically active nitroolefin **127** was started with lipase-mediated acetylation and conversion of the second -OH group to the amide under Mitsunobu conditions, achieving an optically pure product (95% ee) and preventing racemization. The cyclic acetate **124** was then obtained after dehydration of the hemiaminal (Scheme 48).

**Scheme 49:**
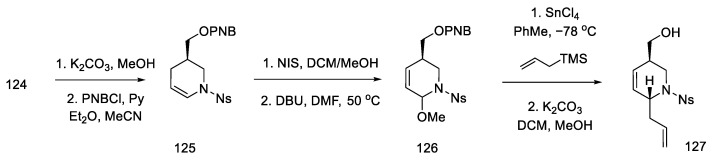
Introducing an allylic system into molecule **126**.

Acetate **124** was converted to the corresponding p-nitrobenzoate enamide **125**, which was recrystallized from methanol. Enamide **125** was then treated with NIS in methanol, followed by dehydrogenation with DBU to give methoxy-olefin **126**. Stereoselective attachment of the side chain was performed by treatment with allyltrimethylsilane (Scheme 49).

**Scheme 50:**
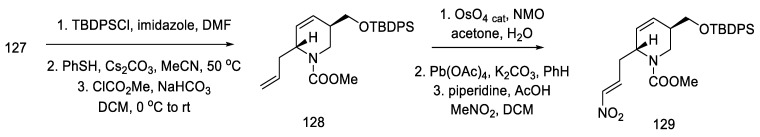
Preparation of nitroolefin **129**.

After modifying the protecting groups, nitroolefin **127** was obtained in two steps by reacting the formed aldehyde with nitromethane (Scheme 50).

**Scheme 51:**
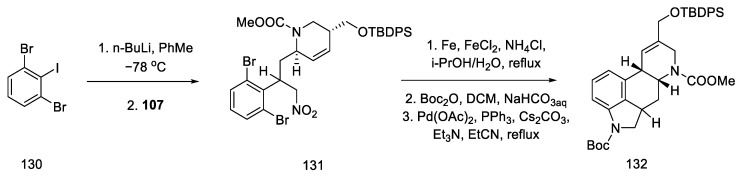
Forming the B and C rings in Fukuyama strategy II.

By using nitroolefin **129**, the key cyclization reactions leading to the tetracyclic ergoline structure could be performed. Synthesis was continued with the formation of an organolithium compound from dibromoiodobenzene and coupling it with a nitroolefin to form the bicyclic compound **131**. Then, using the Heck coupling and intramolecular amine cyclization, two new rings—B and C—were formed, yielding structure **132** (Scheme 51).

**Scheme 52:**
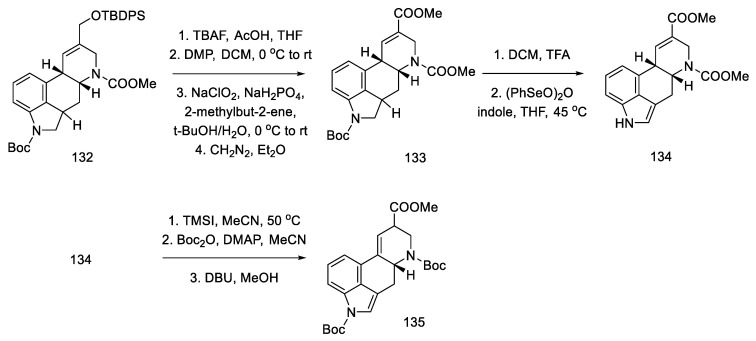
Preparation of a protected lysergic acid ester **135** by Fukuyama strategy II.

The next steps were the manipulation of the protecting groups to give the diester **134** and migration of the double bond from the C8–C9 position to the C9–C10 position using DBU (Scheme 52) [87].

**Scheme 53:**
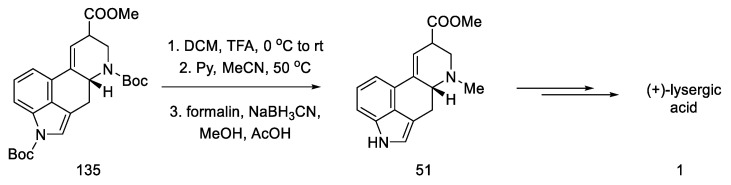
Deprotection and methylation of lysergic acid ester **51**.

Finally, gradual deprotection of both Boc groups and the reductive methylation of dehydropiperidine yielded lysergic acid methyl ester **51** as a mixture of diastereoisomers, with spectroscopic data that were identical to those described in the literature [70]. This compound can be converted into (+)-lysergic acid, according to the procedure proposed by Szantay (Scheme 53) [70].

In conclusion, an asymmetric synthesis of the (+)-lysergic acid ester was achieved using the Heck coupling reaction to close the C-ring of the ergoline structure. The chiral center was introduced in the first step, thanks to the lipase reaction. Although compound **51** is obtained in a long sequence (analogous to the above work), the total yield is 1.25%, which gives an average yield of 88% for a step.

**Scheme 54:**
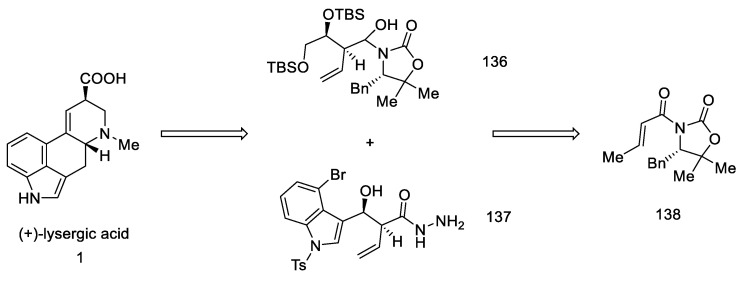
Retrosynthetic analysis leading to substrates in Fukuyama strategy III.

**Strategy III.** Another synthetic attempt to obtain lysergic acid was undertaken by Fukuyama’s team in 2013 [88]. The acid could be prepared from units with stereogenic centers in allylic positions (**136**, **137**), followed by a D-ring closing metathesis reaction, and finally a Heck reaction to close the C-ring. Synthesis was started with what is known in the literature as crotoylooxazolidinone **138** (Scheme 54) [89].

**Scheme 55:**
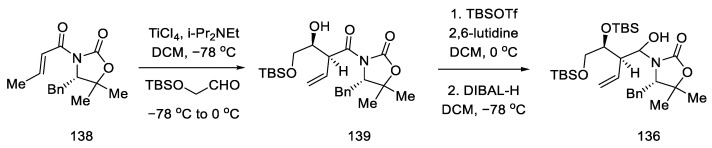
Preparation of the hemiaminal **136**.

Compound **138** was prepared, which was in fact an aldehyde masked by oxazolidine. An Evans aldol reaction was used to obtain an amide with a terminal olefin **139**. After protection of the -OH groups with TBS, reduction of DIBAL-H was performed to give the hemiaminal **136** (Scheme 55).

**Scheme 56:**
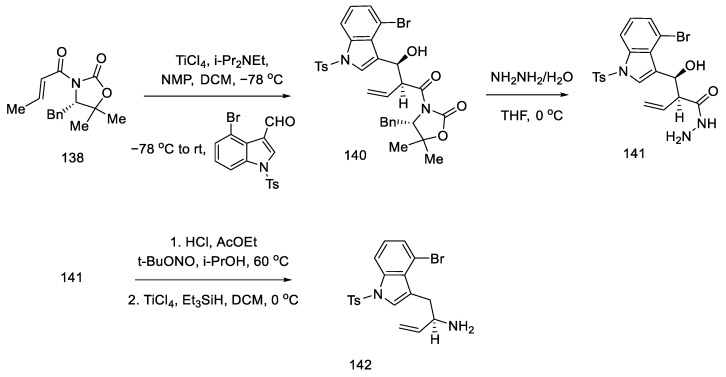
Preparation of allylamine **142**.

The preparation of the second starting material was carried out using the same compound **138**. The Evans reaction was performed again—taking advantage of the fact that this aldol reaction allows stereochemical control at the allylic position—using indole aldehyde, since it is known in the literature. Thereafter, treatment with hydrazine gave hydrazide **141**, which was converted to the acyl azide. Curtius rearrangement of the azide under acidic conditions gave amine which, upon reduction with triethylsilane, gave allylamine **142** (Scheme 56).

**Scheme 57:**
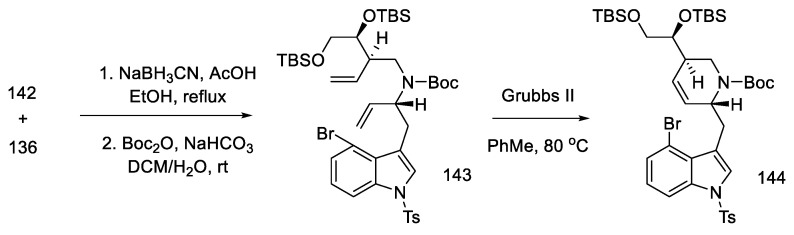
Reaction of allylamine **142** and the hemiaminal **136**, along with reduction by Grubbs catalyst II.

Having both compounds—allylamine **142** and the hemiaminal **136**—reductive alkylation was performed. The amine was then protected with -Boc, and the D ring was closed using a ring-closing double-bond metathesis reaction. The second-generation Grubbs catalyst was used in this reaction [90], which made it possible to perform cyclization to compound **144**, with high efficiency (Scheme 57).

**Scheme 58:**
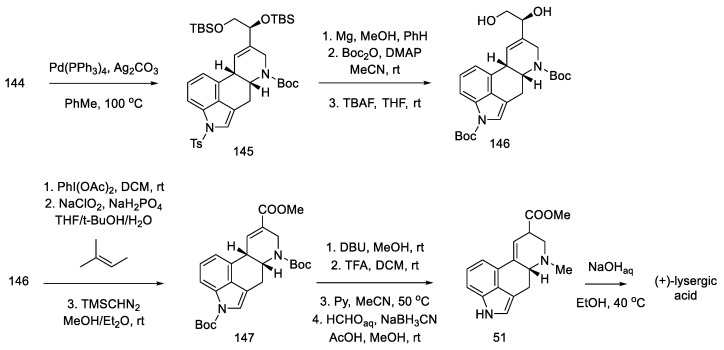
Preparation of lysergic acid in Fukuyama strategy III.

Closing the D-ring using the Heck method yielded tetracyclic product **145**, with an ergoline skeleton. Subsequently, the manipulation of protecting groups and functional groups gave ester **51** and then (+)-lysergic acid, analogous to strategies I and II (Scheme 58). Two successive cyclization reactions were the key steps in this procedure.

**Strategy IV**. The Fukuyama team’s recent work deviates from the idea of using the Heck reaction and instead proposes the formation of a C–C bond between C10 and C11 by aziridine ring cleavage [91]. The reaction begins with the preparation of the substrate—an indoline derivative [92]. Studies have shown that indole undergoes side reactions as a result of knitting the electron-rich 3 position in the bicyclic structure. The choice of the chiral substituent—(3R, 5R, 10R)-trans-aziridine—allowed for the performance of diastereoselective reactions.

**Scheme 59:**
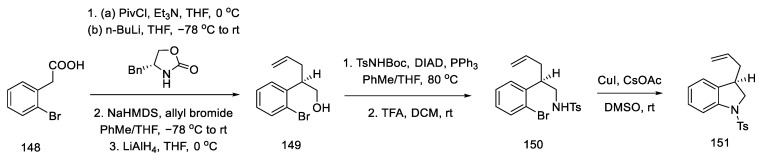
Beginning of the synthesis according to Fukuyama strategy IV.

The synthesis of the allyl indoline derivative started with 2-bromophenylacetic acid **148**. Installation of the chiral auxiliary, followed by allylation and reduction of LiAlH_4_, gave the alcohol **149**. The Mitsunobu reaction was then performed followed by deprotection of the Boc group. The closing of the five-membered cycle of indoline **151** was due to the amination reaction in the presence of copper salts (Scheme 59).

**Scheme 60:**
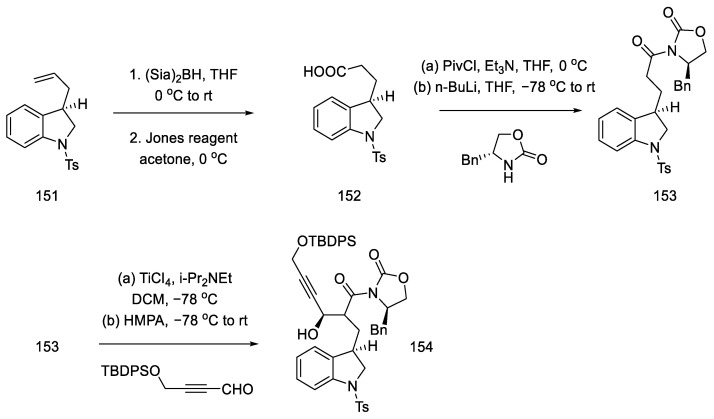
Preparation of alkyne **154**.

Terminal olefin **151** was oxidized to carboxylic acid **152** by treatment with the Jones reagent. The chiral benzyloxazolidin-2-one was reconnected, and a diastereoselective aldol reaction was performed by reacting acyloxazolidinone **153** and an alkyne aldehyde in the presence of a titanium chloride (Scheme 60).

**Scheme 61:**
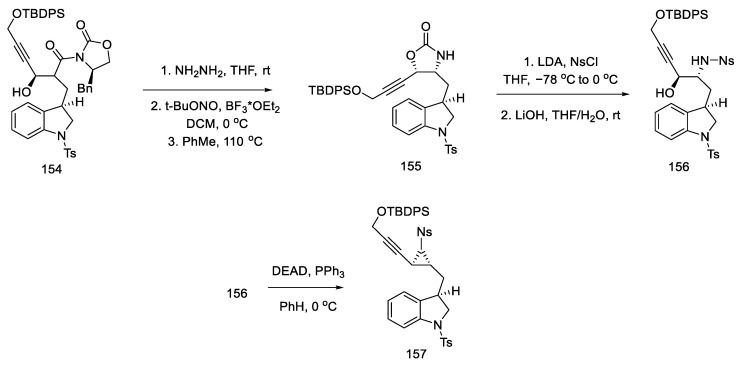
Synthesis of aziridine **157** in the Mitsunobu reaction.

The obtained compound **154** was converted to hydrazide using hydrazine and then reacted with nitrite. The formed acyl azide was used for Curtius rearrangement, analogous to the sequence of reactions described in the previous publication, to give oxazolidinone **155** [88]. The necessary stereogenic centers were, thus, incorporated into the compound. In the next step, a nosyl group was inserted in place of the oxazolidinone, which, upon the addition of lithium hydroxide, gave hydroxy-p-Ns-amide **156**. The Mitsunobu reaction followed, and trans-aziridine **157** was obtained (Scheme 61).

**Scheme 62:**
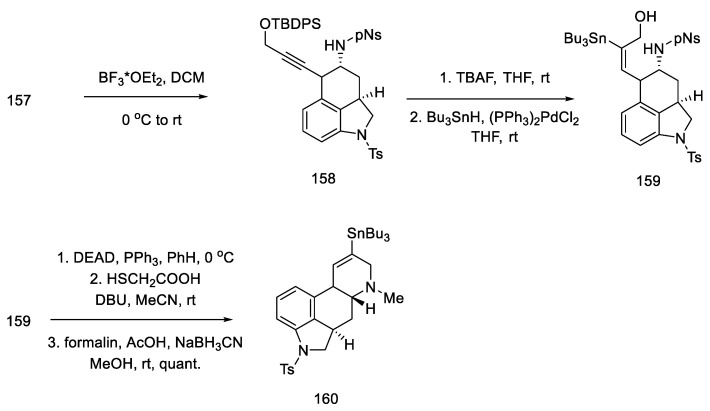
Closing the D-ring in Fukuyama strategy IV.

Upon treatment of aziridine **157** with Lewis acid, the three-membered ring was cleaved stereospecifically. A tricyclic compound containing ABC rings of the ergoline system was obtained. The structure **158** was introduced with an organotin substituent. Upon its use, the D ring was closed by an intramolecular Mitsunobu reaction. The amine was released from the -Ns protecting group with thioglycol acid, followed by methylation with formalin and sodium cyanoborohydride, to give the tetracyclic compound **160** (Scheme 62).

**Scheme 63:**
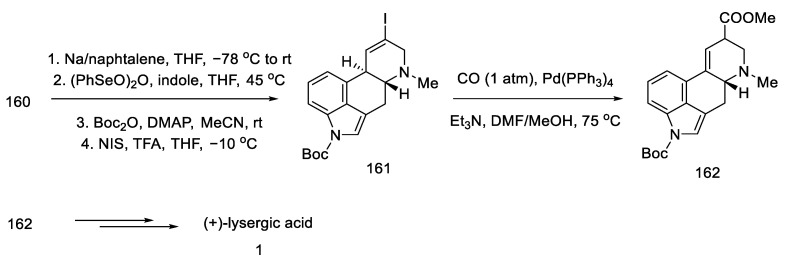
Preparation of lysergic acid in Fukuyama strategy IV.

After manipulation with protecting groups, indoline **160** was oxidized to indole with (PhSeO)_2_O. The organotin substituent was replaced with an alkenyl iodide, followed by the incorporation of CO carbon monoxide in the presence of palladium, to obtain an epimeric mixture of esters **162**. These esters can be converted to lysergic acid by the methods presented in the previous publications by the Fukuyama group or by the method proposed in this publication by treatment with TFA, decarboxylation by heating with pyridine, and alkaline hydrolysis (Scheme 63).

Many years of research by the Fukuyama group have led to a number of solutions to the problem of total synthesis of (+)-lysergic acid. All methods are complex syntheses, focused on the gradual preparation of substrates for the C-ring closure reaction, usually the Heck reaction.

#### 2.4.3. Liu and Jia Research

**Strategy I.** The work of Liu and Jia, from 2011, shows a method of obtaining (+)-lysergic acid in which the starting material is the (R)-derivative of indole **164**, as a result of the D ring closing reaction in the double-bond metathesis reaction, followed by the intramolecular Heck reaction [93]. This is a very similar procedure to the one proposed by Fukuyama, but much shorter [86]. The starting indole derivative can be obtained using D-glutamic acid **163** [92].

**Scheme 64:**
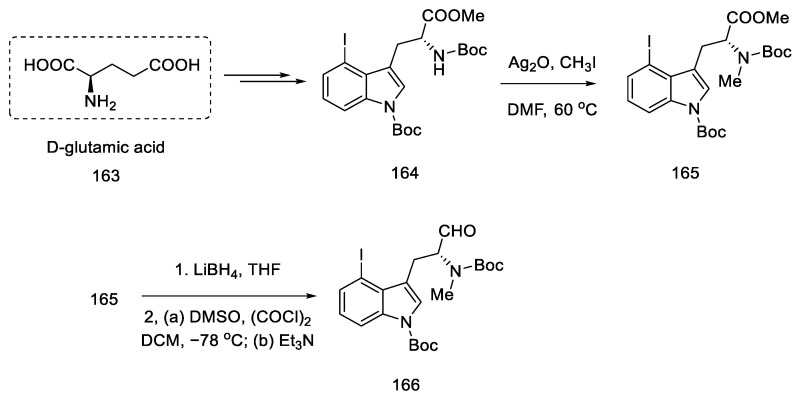
Beginning of the synthesis of lysergic acid from D-glutamic acid **163**.

The synthesis begins with the methylation of the amine with methyl iodide, followed by reduction with borohydride and Swern oxidation. The obtained aldehyde **166** can be used for the Wittig reaction (Scheme 64).

**Scheme 65:**
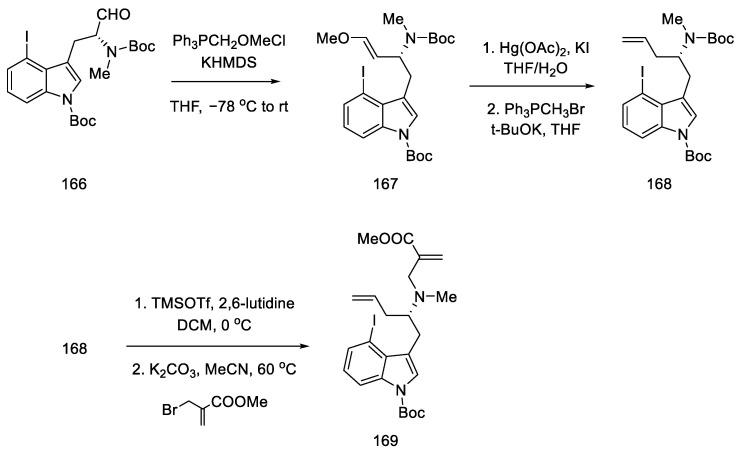
Introducing of two terminal double bonds into molecule **166** in strategy I.

Enol ether **167** was obtained by treatment with methoxymethylene ylide. Successive reactions gave diene **169**, which was a substrate for the D-ring and then C-ring cyclization reaction (Scheme 65).

Compound **169** was treated with a second-generation Grubbs catalyst in the presence of p-TsOH acid [90]. It was tested that, in addition to the presence of a Lewis acid, the choice of solvent (toluene) played a crucial role in the success of the reaction.

**Scheme 66:**
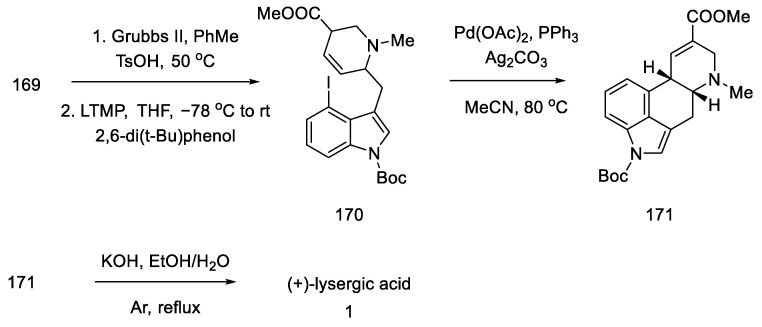
Method of obtaining lysergic acid proposed by Liu and Jia.

Following the synthesis of tricyclic **170**, various C-ring closing pathways were investigated. The γ arylation reaction was unsuccessful, so the Heck reaction was tested. After finding the optimized conditions, the reaction was performed in the presence of two equivalents of silver carbonate in hot MeCN to give compound **171** (Scheme 66).

Finally, the Boc group was deprotected, and ester hydrolysis accompanied by double-bond isomerization was performed to obtain (+)-lysergic acid.

**Strategy II.** The subsequent publication by the Jia team presents an optimized version of strategy I and compares two total syntheses to each other; one is described as strategy I in the above work, plus there is a synthesis in which key stages were swapped (strategy II) [94]. The goal was to further simplify the synthetic path by reducing the effort to protect functional groups. By using 3-chloro-2-iodoaniline, it was possible to simplify the indole coupling to give a compound that could be directly converted into the Heck reaction. The reaction started with simple substrates including chiral (R)-sulfonamide **172**.

**Scheme 67:**
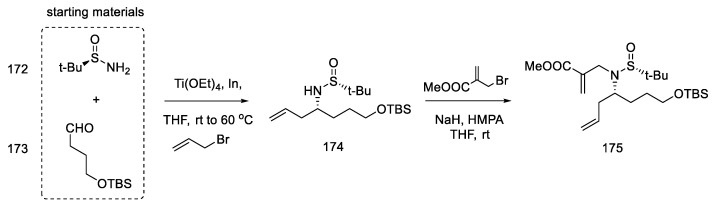
Introducing two terminal double bonds into the molecule **173** in strategy II.

The first step was to construct a diene that could then be converted to a tetrahydropyridine ring by a metathesis reaction. For this purpose, the aldehyde **173**, (R)-N-tert-butanesulfinamide **172**, and butenyl bromide were reacted, and the chiral allylamine **174** was obtained in the presence of indium and titanium (dr = 7:1) [95]. In the next step, the reaction with bromomethylacrylate gave diene isomers **175**, which were separated by column chromatography (Scheme 67).

**Scheme 68:**
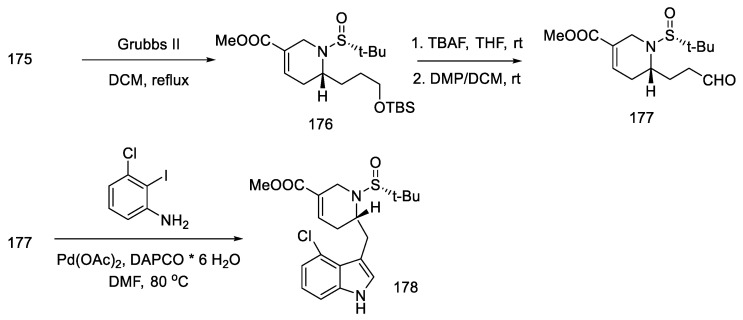
Synthesis of the ABD tricyclic system in Liu and Jia’s strategy.

The metathesis reaction gave the cyclic product **176** by the action of the Grubbs II catalyst in boiling methylene chloride [90]. The -TMS group was deprotected, and the aldehyde **177** was obtained by the Dess–Martin reaction. The obtained product is a fragment of the target structure with the D-ring. The next step was the coupling of the 3-chloro-2-iodoaniline fragment with an aldehyde **177** to synthesize the indole derivative **178** (Scheme 68).

**Scheme 69:**
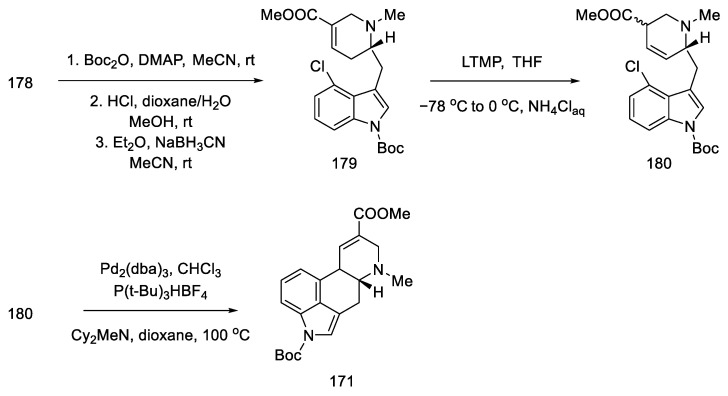
Preparation of lysergic acid precursor **171**.

Further synthesis relied on manipulating the functional groups to obtain substrate **180** for the intramolecular Heck reaction. To this end, the nitrogen atom in indole was protected with the Boc group, then the tert-butanesulfinyl group was removed, and methyl was introduced onto the nitrogen of the D-ring. Double bond isomerization was performed. Optimization of the Heck reaction was performed. Various reaction conditions were tested (palladium sources, ligands, bases, and solvents). The best results were achieved using 0.5 equivalents of Pd_2_(dba)_3_CHCl_3_, 1.0 equivalents of P(t-Bu)_3_HBF_4_, and 3.0 equivalents of Cy_2_MeN in dioxane at 100 °C. Compound **171**, a precursor to lysergic acid, was obtained (Scheme 69). The procedure for obtaining acid therefrom is described in the above works [93].

In two papers presented by the Jia group, two strategies for the preparation of enantioselective acid synthesis of (+)-lysergic acid were described. Both involved metathesis to the D-ring, synthesis of the indole derivative (AB-ring), and the closure of the C-ring by the intramolecular Heck reaction. In strategy I, lysergic acid is obtained in 20 steps. Strategy II allowed for greater convergence of the method and simplified the procedure to just 12 steps.

### 2.5. Other Chemical Methods of Obtaining Lysergic Acid

#### 2.5.1. Oppolzer Group Research

**Scheme 70:**
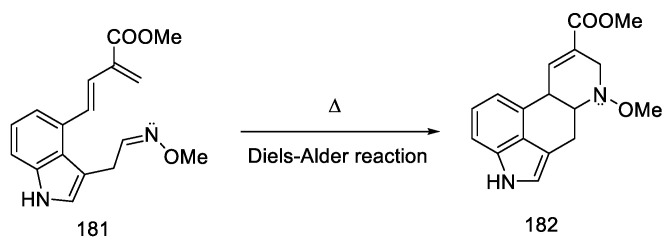
C and D rings closing strategy in the Diels–Alder reaction.

The Oppolzer group presented the synthesis of (±) lysergic acid, where the C and D rings of the ergoline system were formed through an intramolecular Diels–Alder reaction [85]. This is a convergent reaction using protected 4-hydroxymethylindole as a substrate. The synthetic strategy was based on the preparation of an indole derivative substituted with a diene fragment, then connection of the dienophilic system, followed by cyclization (**181** to **182**, Scheme 70) and isomerization of the double bond to the lysergic acid.

The synthesis started with the conversion of indole-alcohol **183** to the corresponding bromide with carbon tetrabromide in the Appel reaction. Then the obtained product was treated with phosphine to obtain phosphonium bromide **184** [96].

**Scheme 71:**
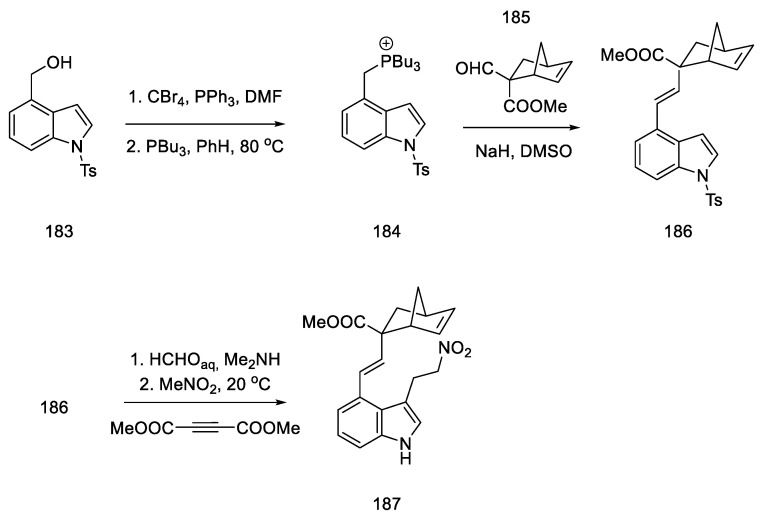
Introduction of a masked diene system into the molecule **184**.

Difficulties were encountered with introduction of the 2-carbomethoxydiene system into the indole derivative. For this purpose, this moiety was masked with the norbornene fragment. The synthesis of the norbornene derivative **185** was performed using conditions from the literature; formylation of methylbicyclo[2.2.1]hept-5-enyl-2-carboxylate with methyl formate in the presence of LDA gave the expected product **185** [31]. Adding compound **185** into the phosphine salt **184** via the Wittig reaction allowed for obtaining a masked diene moiety in product **186**. Then, by nucleophilic addition to nitro compound **187,** a fragment of an electron-poor dienophile was attached to the structure (Scheme 71). This reaction was carried out in two ways—using Michael addition to nitroethylene (24% yield) or in a two-step synthesis—by reacting a Mannich reaction with formaldehyde, followed by adding nitromethane in the presence of acetylenedicarboxylate (48%) [97].

Nitro compound **187** was converted to the oxime **188**. The diene moiety was then revealed by retro Diels–Alder thermal reaction, yielding key intermediate **181** proposed by Oppolzer. The intramolecular reaction required keeping the concentration of the intermediate very low throughout the reaction. The dienophile/diene system generated in situ, thus, underwent a Diels–Alder reaction to give the tetracyclic **182** system, as a mixture of diastereomers.

**Scheme 72:**
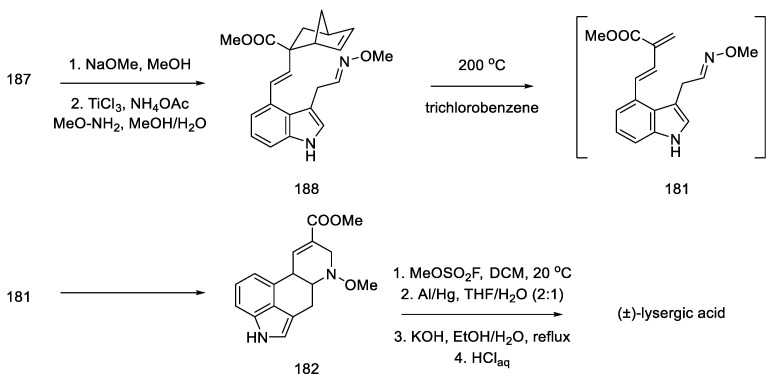
Preparation of lysergic acid by the Oppolzer procedure.

The formal synthesis of lysergic acid was completed by replacing N-methoxy group with methyl group using methyl fluorosulfonate, followed by hydrogenolysis and hydrolysis (Scheme 72). NMR analysis confirmed the identity of the obtained product [58].

#### 2.5.2. Research Carried out by Inuki, Iwata. and Ohno

The group led by Ohno was investigating the preparation of heterocyclic compounds by reaction of allenes. These methods were used to synthesize lysergic acid via two synthetic pathways. The key reactions in these pathways were the preparation of the cumulative double bond system giving the allene, followed by the domino reaction of the palladium-catalyzed cyclization of the allene to form a new stereogenic center. This is due to the transfer of the axial chirality of the C=C=C system to the asymmetric carbon [98,99,100].

**Strategy I.** The synthesis of lysergic acid, lysergol, and isolysergol was carried out. The synthetic strategy was based on the synthesis of allenes by Claisen rearrangement of the corresponding enol ether, followed by cyclization of the CD rings via a palladium-catalyzed domino reaction [98].

**Scheme 73:**
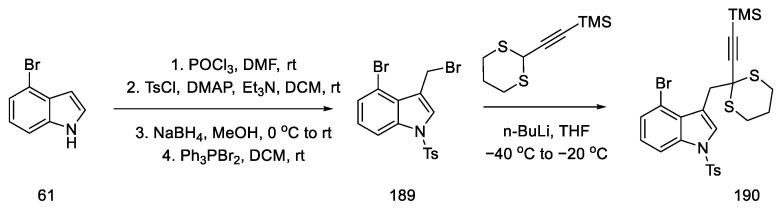
Synthesis of thioacetal **190** from bromoindole.

The preparation of allenes was proposed by starting the synthesis of 4-bromoindole **61**. Reactions from the literature can afford the protected bromomethylindole **189**. Using n-butyllithium, a coupling reaction was performed substituting the indole with acetylenecarbaldehyde, with a source that was thioacetal **190**. Compound **190** was obtained in 96% yield (Scheme 73).

**Scheme 74:**
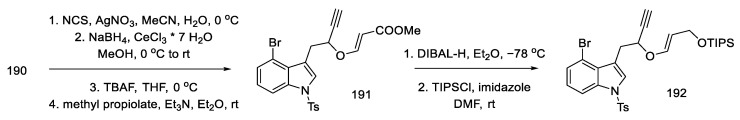
Enolate ether **192** synthesis.

Then, by hydrolysis of the thioacetal **190**, reduction with sodium borohydride, removal of the TMS protecting group, and addition of methyl propiolate, the enol ether **191** was obtained. In the next reaction, the reduction and protection of the alcohol formed with the TIPS-protected group **192** was carried out (Scheme 74). The compound prepared in this way was a substrate for the Claisen rearrangement reaction, leading to the allene **193**.

**Scheme 75:**
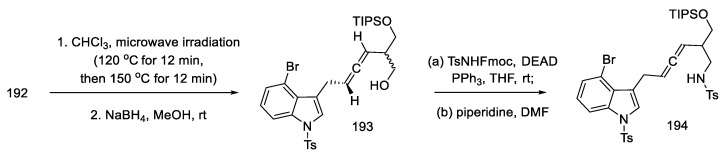
Obtaining allene in strategy I.

Optimizing the conditions led the authors to a procedure that used microwave radiation. A diastereomeric mixture of products **193** was obtained, which was used without further purification. The Mitsunobu reaction with NsNH_2_, analogous with TsNHF_moc_, gave N-nosyl and N-tosyl derivatives **194** (Scheme 75). Better results of the following reactions have been achieved by using a tosyl group.

The preparation of the tetracyclic ergoline structure was accomplished by palladium-catalyzed domino cyclization. The best conditions were selected by testing different ligands and bases. The best yield and diastereomeric excess (dr = 83:17 in favor of the desired diastereomer) was obtained with 5% mol Pd(PPh_3_)_4_ and 3 eq of potassium carbonate in hot DMF.

**Scheme 76:**
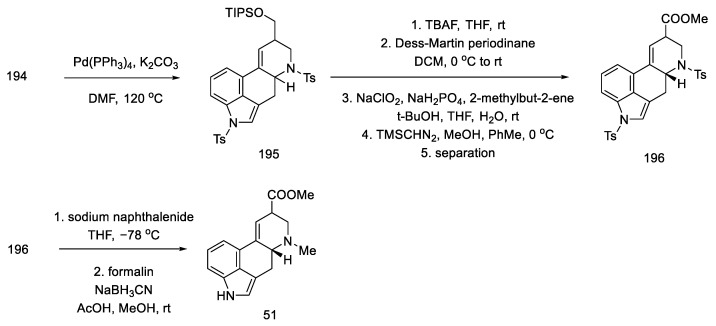
Preparation of lysergic acid ester **51** proposed by Inuki, Iwata, and Ohno.

Tosyl-protected compound **195** was a lysergic acid precursor. Cleavage of the TIPS group, oxidation of the resulting alcohol in the Dess–Martin procedure, and esterification gave the product **196.** Then, the NH groups were deprotected, and the ester **51** used in the synthesis of lysergic acid was obtained (Scheme 76), which can be converted into the desired, optically pure acid by alkaline and acid hydrolysis.

**Strategy II.** The allene was obtained by the reductive coupling reaction of aziridine palladium and the creation of the C-C bond between the indole derivative and oxazinane, followed by the Myers reaction [100].

**Scheme 77:**
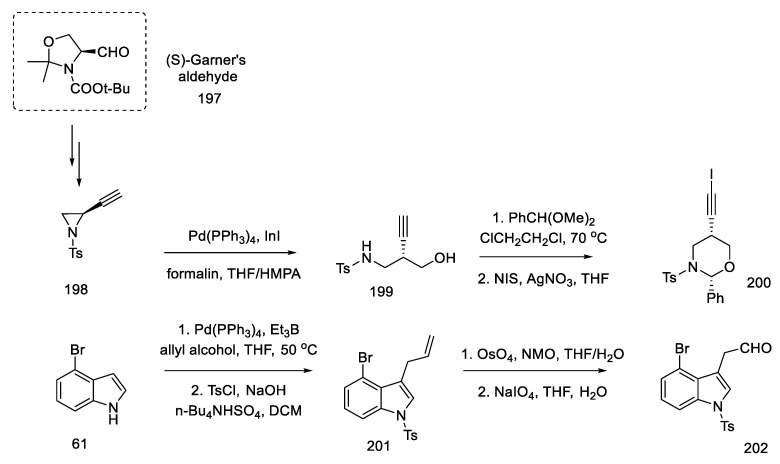
Synthesis of materials for the coupling in strategy II.

First, the materials for the coupling were prepared. For this purpose, bromoindole aldehyde **202** was prepared from bromoindole **61**, using the coupling reaction of allyl alcohol according to the procedure in the literature [101], protection of the nitrogen atom in the indole, and then oxidative cleavage of olefin **201** to aldehyde **202**. The second substrate was the chiral oxazinane derivative **200**. It was prepared from optically pure aziridine **198** (97% ee), which, in turn, is obtainable from (S)-Garner aldehyde **197** in a four-step synthesis [102]. By treating aziridine **198** with a catalytic amount of a palladium compound with InI in a mixture of THF/HMPA and formalin, the amino alcohol **199** was obtained, which could be easily changed into the oxazinane derivative **200** (Scheme 77). Both iodinated and free terminal alkyne enable the coupling reaction to be carried out; however, by introducing iodine, the efficiency and selectivity of the coupling process increase.

**Scheme 78:**
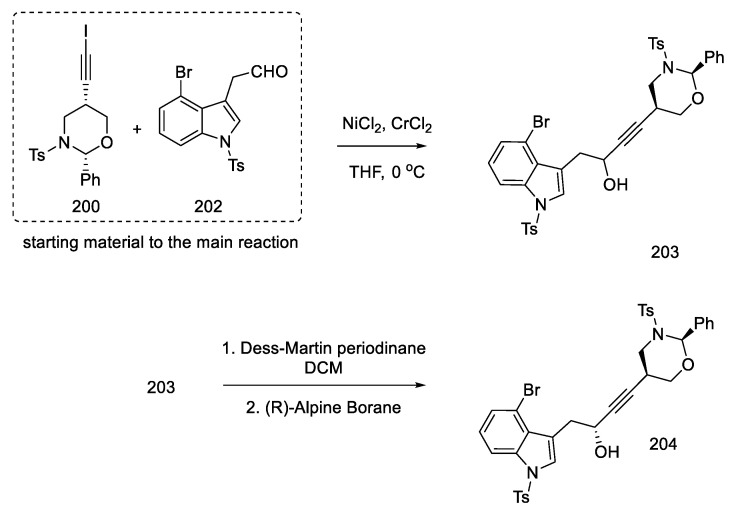
Reaction leads to an asymmetric alcohol **204**.

With the prepared substrates **200** and **202** in hand, the process of their combination was started. The coupling reaction of these fragments was carried out using nickel chloride and chromium chloride. A racemic mixture of alcohol **203** was obtained. Dess–Martin oxidation followed by reduction with (R)-Alpine Borane made it possible to obtain the desired asymmetric product **204** from the racemic alcohol (dr = 95:5) (Scheme 78).

**Scheme 79:**
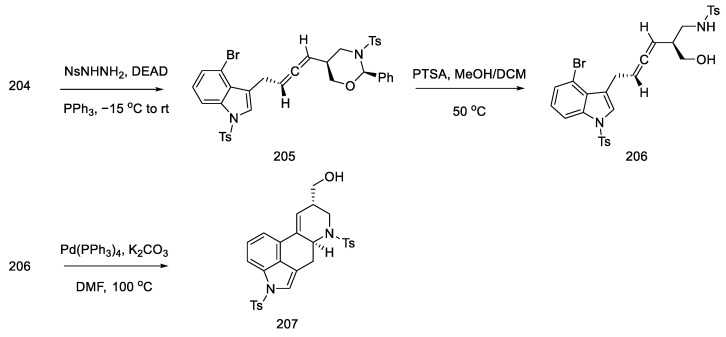
Obtaining allene in strategy II.

Alcohol **204** was stereoselectively converted to the allene **205** by the Myers method [103], using nitrobenzenesulfonyl hydrazine under Mitsunobu conditions. Then the oxazinane was cleaved with PTSA. The product **206** was obtained with a diastereomeric excess (94:6), but the mixture was not separated. A domino cyclization reaction through palladium compounds was performed under optimized conditions (5 mol% Pd(PPh_3_)_4_, K_2_CO_3_, and DMF at 100 °C). The analogous reaction for the second epimeric product was also checked using reduction with (S)-borane. The cyclization reaction from the allene gave the corresponding diastereoisomer **207** with high selectivity. This compound is the key intermediate in the approach used by the Ohno group (Scheme 79).

**Scheme 80:**
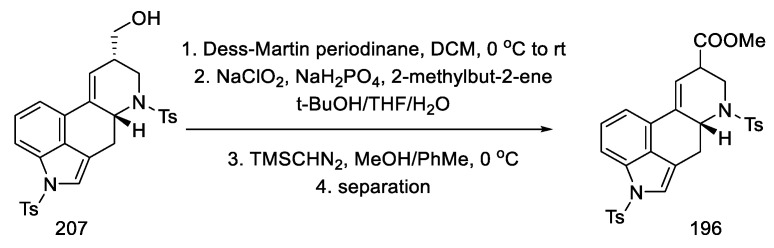
Obtaining intermediate **196** in strategy II.

The primary alcohol **207** was oxidized with a Dess–Martin reagent and chlorite. The esterification was then carried out to the known racemic methyl ester, of which both amine functions were protected with tosyl (**196**), which could be transformed into lysergic acid (Scheme 80) [64,70,98].

The above publication describes methods for the synthesis of other ergot alkaloids, lysergol and isolysergol. The total synthesis of lysergic acid consists of 19 steps, and the overall yield is 1.1%. The starting material was Garner’s aldehyde. The innovation of this synthetic strategy involves the construction of the D ring as a result of catalytic cyclization of the allene. Further work by Ohno’s team boiled down to new methods of obtaining the above allene, which can then be easily transformed into the intermediate **51.**

**Strategy III.** Strategy III of obtaining lysergic acid—specifically of intermediate **51**—was based on similar assumptions as strategy II. The bromoindole derivative **61** and the corresponding alkyne were prepared, and the coupling reaction was performed. The resulting alkyne was converted to an allene, which could be converted to the corresponding intermediate. The new idea was to obtain a chiral alcohol, not by an asymmetric oxidation reaction, but by a regioselective epoxide opening sequence. For this purpose, the reactants were modified—the synthesis started with bromoindole and the corresponding sulfonamide terminal (S)-alkyne, as known in the literature [99].

**Scheme 81:**
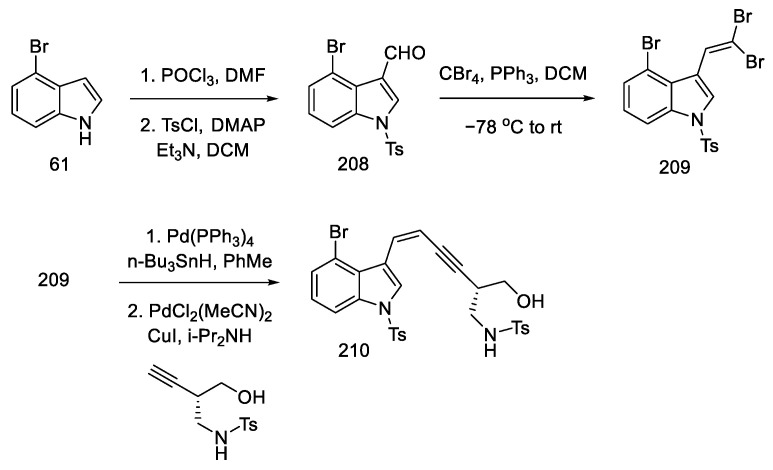
Synthesis of enyne **210** from bromoindole.

Commercially available 4-bromoindole **61** was formylated by the Vilsmeier–Haack reaction [104], followed by treatment of resulting aldehyde **208** with CBr_4_, which gave dibromoolefin **209**. Using the Uenishi method [105], followed by the Sonogashira coupling of the known chiral alkyne, which can be prepared from (S)-Garner aldehyde **197** (analogous to strategy I), a compound was obtained containing a triple and a double bond at adjacent carbon atoms—enyne **210** (Scheme 81).

**Scheme 82:**
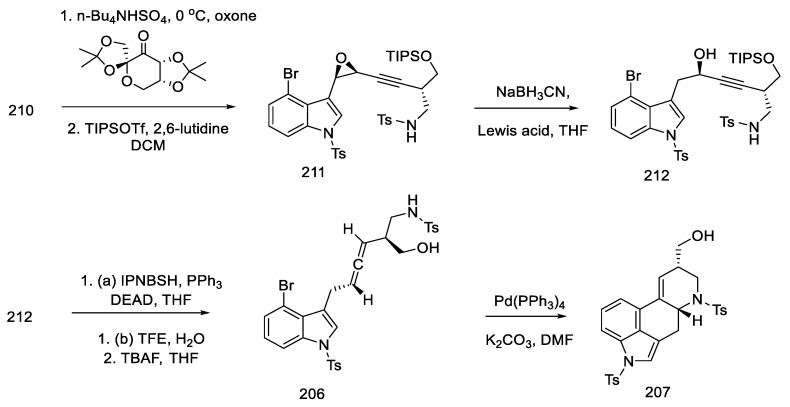
Obtaining allene in strategy III.

The epoxidation of enyne **210** was a challenging step. Various epoxidizing agents were tested, but neither the use of the Shi catalyst nor the m-CPBA acid allowed for obtaining the epoxide **211** in a satisfactory yield. Only the optimization of the conditions (presence of n Bu_4_NHSO_4_, oxone) allowed the yield of the product to be over 70%, with a diastereomeric ratio of 82:18. The OH group was protected with TIPSOT, and then chiral alcohol **212** was obtained by ring opening with Lewis acid (ZnCl_2_ or Zn(OTf)_2_), retaining the excess diastereoisomer. Alcohol **212** was converted (analogycal to strategy II) in allene **206** using a modified Myers reaction via nitrobenzenesulfonyl hydrazine (Scheme 82) [106].

In summary, strategy III used the following reactions: obtaining a bromoolefin, a derivative of indole **209**; then, via the Uenishi reaction and Sonogashira coupling to a terminal alkyne, the enyne, thus obtained, was oxidized by an asymmetric epoxidation reaction, and the alkene oxide was opened by regioselective reduction to the chiral alcohol. This alcohol, with a carbon–carbon triple bond in its structure, was converted into an allene in a modified Myers reaction, which, analogically to strategy I and II, can be converted into intermediate **51**, and, further, into (+)-lysergic acid.

### 2.6. Attempts of Obtaining Lysergic Acid by Other Methods

#### 2.6.1. Padwa Group Research

In 1995, the Albert Padwa research group developed a new synthetic pathway that could eventually lead to lysergic acid [107].

**Scheme 83:**
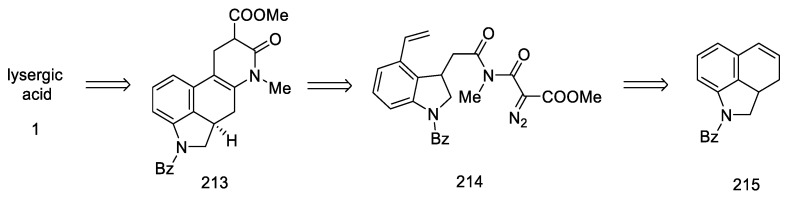
Retrosynthetic analysis proposed by Padwa.

First, it was shown that the intramolecular dipolar cycloaddition of isomuenchnone by an olefin or an alkyne leads to the quinoline structure—the C and D ring of ergot alkaloids. Subsequently, a 12-step synthesis was carried out, starting from the known tricyclic olefin **215**, through diazoimide **214,** to lysergic acid derivative **213** (Scheme 83). Unfortunately, the double-bond isomerization, which would lead to a lysergic acid structure, could not be carried out.

**Scheme 84:**
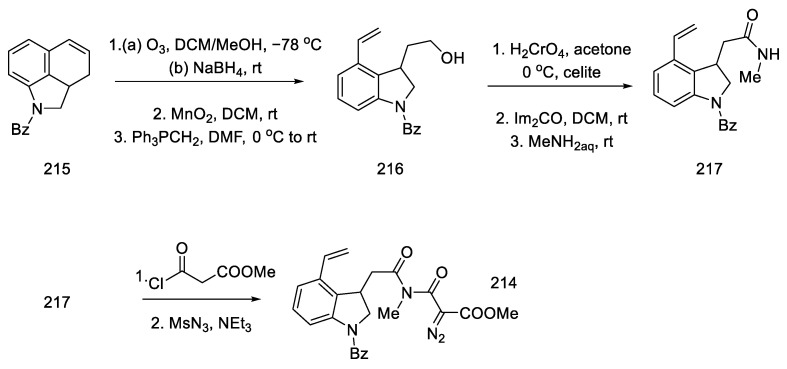
Beginning of the synthesis proposed by Padwa.

The synthesis began with the known protected tetrahydrobenzoindole **215**. Ozonolysis was performed followed by reduction with sodium borohydride. A diol was obtained, which was selectively oxidized, followed by a Wittig reaction, to give olefin **216**. In the next three steps, amide **217** was obtained from the olefin using the Jones reagent, carbonyldiimidazole, and methylamine. Diazoimide **214** was prepared by the action of acid chloride and azide. This compound was used to study the cyclization reaction using the Rh(II) catalyst (Scheme 84) [108].

The cyclization conditions have been optimized by selecting the best catalyst. The best selectivity was achieved using perfluorobutyrate (pfb) as the rhodium catalyst ligand.

**Scheme 85:**
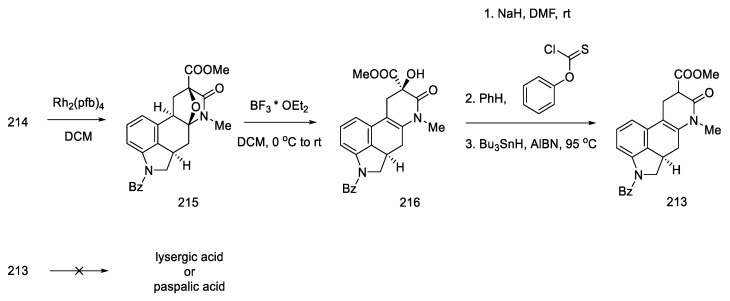
Continuation of the synthesis proposed by Padwa.

The conversion of the oxygen bridge containing product **215** to the corresponding enamide **216** was performed using boron fluoride as a Lewis acid. The enamide, thus obtained, was treated with thiocarbonate in benzene by performing the Barton–McCombie reaction [109]. Chiral ester **213** was obtained in the form of a mixture of diastereoisomers. Unfortunately, all attempts to isomerize the double bond have been unsuccessful (Scheme 85).

In conclusion, the total synthesis of ergot alkaloids—lysergic acid and paspalic acid—could not be completed. At the same time, the method proposed by Padwa allows for the closure of the C and D rings to the ergoline structure, and further research may lead to the achievement of the assumed goal.

#### 2.6.2. Parsons Group Research

One year after the Padwa research, an article by the Parsons group was published, which presented the results of new methods of performing tandem reactions for the ring closure in natural compounds and their analogues [110]. This publication summarizes the team’s achievements, including the possibility of closing three- and four-membered systems leading to ergoline, as presented previously [111]. There are also studies conducted by Ȍzlȕ and Cladingboel, who attempted to synthesize lysergic acid [112]. In these studies, free radical reactions obtained by homolytic cleavage of a carbon–bromine bond with a hydride were used. Radical reactions allow single or double (tandem) cyclization reaction to take place.

The starting material was 2-bromo-3-nitrobenzoic acid **217**. As a result of the multistage synthesis, the aldehyde **218** was obtained, which was then reacted with amines to give compounds **219** and **221**.

Cladingboel proposed a radical cyclization reaction using MPM-protected compound **219**. Tandem cyclization, performed with tributyltin hydride and AIBN, resulted in reduced to a five-membered structure of the D-ring **220**.

**Scheme 86:**
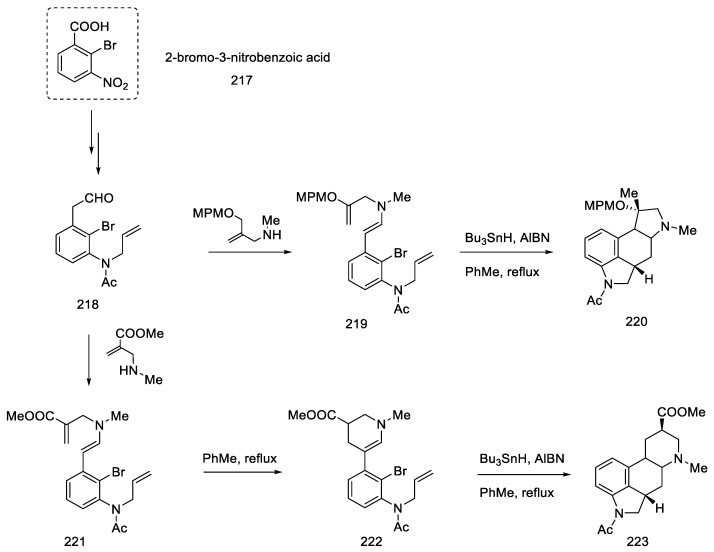
Parsons group synthetic strategies.

Continuing this work, Ȍzlȕ used methyl 2-(methylamine)acrylate to introduction an ester group in compound **221**. Operating at elevated temperature, cyclization of the six-membered D-ring was performed; however, the radical reaction also led not to lysergic acid but to its saturated derivative **223** (Scheme 86).

### 2.7. Biosynthetic Pathways of Lysergic Acid

The pioneer of the topic of ergot alkaloids biosynthesis was Floss, who, in 1976, presented a set of information on the synthesis processes in biological systems. Since then, many processes have been clarified and the related knowledge has been expanded [113].

A study by Shu-Ming Li’s team, published in 2014, outlines the biochemical pathways leading to ergot alkaloids, including lysergic acid [9]. The study collected information on intermediates, the enzymes used as biocatalysts, and the genes that allowed specific reactions to take place. Not all the mechanisms have been confirmed, but the identification of intermediates allows for understanding the most likely reaction mechanisms. Similarly, Mukherjee and Joydeep, in 2000, put together strategies to chemically and biochemically synthesize ergot alkaloids [114].

**Scheme 87:**
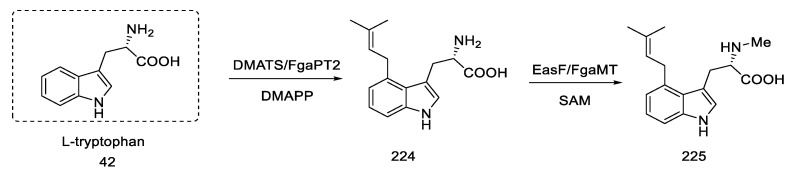
The first stages of lysergic acid biosynthesis.

Lysergic acid biosynthesis begins with L-tryptophan. In the first step, C4-prenylation was carried out with the use of an enzyme isolated from *Claviceps* sp., which is encoded by the *dmaW* gene [115]. For this purpose, the biochemical reaction takes place with dimethylallyl diphosphate as a prenyl donor, in the presence of a biocatalyst—DMATS synthase prenyltransferase [116]. The mechanism of this reaction, proposed by Metzger, resulted from the X-ray structure of the active complex. It includes three stages: formation of the dimethylallyl cation, nucleophilic attack of the indole nucleus to the cation, and deprotonation [117]. The second step in biosynthesis is N-methylation of allyl-tryptophan **224** to the L-abrine derivative **225**, catalyzed by EasF methyltransferase in the presence of (S)-adenosylmethionine SAM (Scheme 87) [118].

**Scheme 88:**
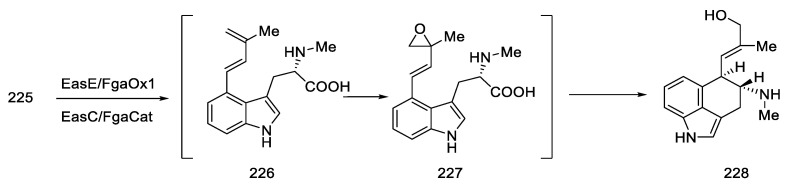
Biomechanism proposed by Metzger.

The mechanism of conversion of compound **225** to **228** is not confirmed. It is known that this reaction requires decarboxylation and oxidation steps using at least two enzymes—FAD-dependent oxidoreductase (FgaOx1) and FgaCat catalase, in which *easE* and *EasC* genes play a key role. Presumably, this process occurs through the steps of: desaturation of the C8–C9 bond by hydroxylation of the benzyl carbon, deprotonation of the C17 carbon, oxidation of the C7–C8 bond, and decarboxylation of the epoxide **227** intermediate (Scheme 88) [119,120,121,122].

**Scheme 89:**
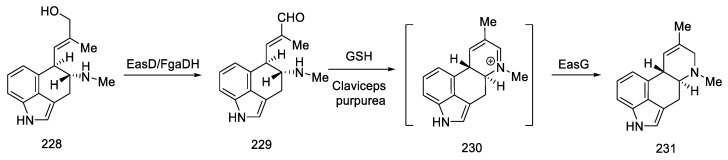
Enzymatic cyclization of the D-ring.

The next step in the synthesis leads to the aldehyde **229**. It is obtained by a short-chain dehydrogenase/reductase-catalyzed reaction SDR. Wallwey used FgaDH for this purpose in the presence of NAD+ [123]. Aldehyde **229** is an important intermediate, from which compounds such as festuclavine, pyroclavine, and agroclavine can be obtained through the biochemical pathway of fungi. The four-ring agroclavine **231** can be obtained from *Claviceps purpurea* by treating the aldehyde of the enzyme from *E. festucae* var. lolii or *EasG*, as a result of which an adduct with reduced glutathione is transformed into the desired intermediate (Scheme 89) [124,125].

**Scheme 90:**
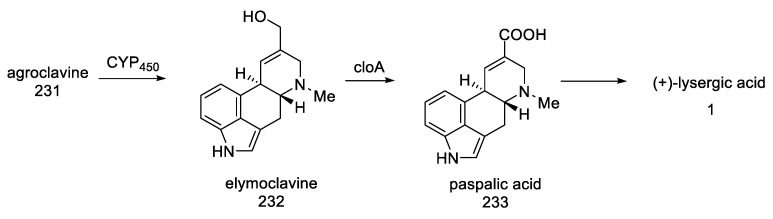
Preparation of lysergic acid from agroclavine.

The biosynthesis of lysergic acid from agroclavine **231** remains unexplained. Among the intermediates leading to the final product, elymoclavine **232** and paspalic acid **233** can be distinguished. It is postulated that cytochrome P-450 monooxygenase, or rather its gene—*cloA*, is an essential enzyme for subsequent transitions. Elimoclavin can be obtained by the two-electron oxidation of agroclavin [126]. Then the application of four-electron reduction leads to paspalic acid **233** [126,127]. Paspalic acid can be converted to lysergic acid either by the action of isomerase or by spontaneous rearrangements (Scheme 90) [113].

The genes for making ergot alkaloids are grouped together in the producer’s genomes. By genetic modification of the host, the amount and type of ergot alkaloids produced can be varied [128].

**Scheme 91:**
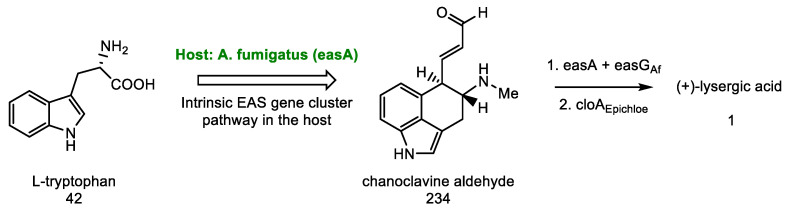
Lysergic acid biosynthesis strategy proposed by Zhu.

At this point, it is worth mentioning the publication “Recent progress in ergot alkaloid research” by Ping Zhu [129]. In addition to proposing the mechanisms of action of some ergot alkaloids, they presented the characteristics of the key genes and gene clusters involved in the biosynthetic process leading, inter alia, to lysergic acid. In particular, the focus was on genetically modifying the strains, so that the main acid and its derivatives can be produced through a biological engineering strategy (Scheme 91).

An exemplary strategy is to start the biosynthesis with L-tryptophan **42** and DMAPP. Next, in the modified host organism, there are transformations from chanoclavine to aldehyde **234**, and then through agroclavine to lysergic acid [130].

As mentioned at the beginning, the annual production of lysergic acid is over 10 tons per year. Chemical total syntheses are characterized by low total yields; therefore, biotechnological methods are implemented. In 2022, an analogous biosynthetic pathway (starting with tryptophan), but using a modified yeast chassis of the DDM33B strain, was used to investigate the amount of acid that could be obtained from a biotechnological batch culture. The scale-up studies allowed for estimating that 52 g of lysergic acid can be obtained per year using the 1000 L bioreactor [131].

## 3. Conclusions

Lysergic acid is a very important organic compound that is the common unit of ergot alkaloids. Its intriguing nature has attracted the attention of many research groups. Therefore, the study of its properties and methods of synthesis has been carried out for many decades. In the above work, with the best knowledge of the authors, all known chemical methods of obtaining lysergic acid have been described. Methods leading to its synthesis, through Woodward, Hendrickson, and Szantay intermediates and Heck coupling methods, are presented. Other synthetic methods are also summarized, and attempts, although unsuccessful, are described, which can be elaborated in the future for the preparation of the ergoline system and related derivatives. The biosynthesis of lysergic acid has also been described. Different approaches, throughout the years, to lysergic acid have been summarized in Table 2. Lysergic acid does not cease to inspire new generations of researchers. We are likely to see novel ideas emerging, so this beautiful molecule will see solutions to old problems as well as completely new approaches that are yet to be discovered.

## Figures and Tables

**Table 1:** Activity profile of ergot alkaloids—lysergic acid derivatives.

Lysergic Acid Derivatives	Ergotism	Uterotonic Contraction	Vasoconstriction
Ergotamine	Very strong effect	Strong effect	Very strong effect
Dihydroergotamine	Moderate effect	Moderate effect	Strong effect
Lysergic acid amides ^1^	Moderate effect	Very strong effect	Moderate effect
LSD	Strong effect	Strong effect	Moderate effect

^1^ ergometrine and methylergometrine.

**Table 2:** Summary of chemical methods of obtaining lysergic acid.

Name of Strategy	Year	Key Intermediate	Optical Rotation	Steps *	Yield [%] **	Ref
**Woodward strategy**		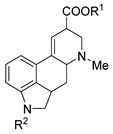	(±)			
Woodward	1956			15	0.8	[31]
Julia (Baillarge group)	1969			11	-	[47]
Ramage	1976			19	1.5	[32]
Kiguchi and Ninomiya	1982			20	0.03	[59]
Rebek	1983			14	4.4	[55,56]
**Hendrickson strategy**		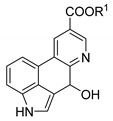	(±)			
Hendrickson	2004			8	10.6	[64]
Yigang (Snieckus group)	2010			9	-	[67,69]
Beaundry	2020			10	-	[68]
**Szantay strategy**	-	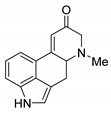	(+)			
Szantay	2004			15	0.7	[70]
Garner	2021			20	0.21	[74]
**Closing the C/D-ring** **using the Heck method**						
Ortar	1986		(±)	14	2.0	[79]
Kurihara	1988		(±)	12	1.3	[80,81]
Fukuyama	2009		(+)	34	0.9	[80]
Fukuyama	2009		(+)	24	0.08	[86]
Fukuyama	2013		(+)	19	12	[88]
Fukuyama	2018		(+)	30	0.07	[91]
Jia and Liu	2011		(+)	20	5.1	[93]
Jia and Liu	2013		(+)	12	1.0	[94]
**Other**						
Oppolzer	1981		(±)	17	0.9	[85]
Fuji and Ohno	2008		(±)	21	3.1	[98]
Fuji and Ohno	2011		(+)	16	5.9	[99]
Fuji and Ohno	2011		(+)	17	1.8	[100]

* longest linear sequence from commercially available precursor to lysergic acid. ** where it was possible, combined reported yields of reactions in every step.

## Data Availability

Not applicable.

## Abbreviations

Ac	acetyl
AlBN	azobisisobutyronitrile
Alloc	allyloxycarbonyl
Aq	aqueous
BC	before Christ
Bn	benzyl
Boc	tert-Butyloxycarbonyl
Bz	benzoyl
°C	degree Celsius
Cat.	catalyst
Cbz	benzyloxycarbonyl
Cp	cyclopentadienyl
CSA	camphorsulfonic acid
Cy	cyclohexane
DAPCO	1,4-diazabicyclo[2.2.2]octane
DBU	1,8-diazabicyclo(5.4.0)undec-7-ene
DCE	1,2-dichloroethane
DCM	dichloromethane or methylene chloride
DEAD	diethyl azodicarboxylate
DEPC	diethyl pyrocarbonate
DIAD	diisopropyl azodicarboxylate
DIBAL-H	diisobutylaluminium hydride
DMAP	4-Dimethylaminopyridine (less frequently used for 4-Dimethylaminophenol)
DMAPP	dimethylallyl pyrophosphate
DMATS	dimethylallyl tryptophan synthase
DMF	dimethylformamide
DMSO	dimethyl sulfoxide
*E. coli*	lat. *Escherichia coli*
EAS	5-epi-aristolochene synthase
e.g.	lat. *exempli gratia*, for example
Et	ethyl
Fmoc	fluorenylmethoxycarbonyl
Fr.	short name of discoverer (botanist) Elias Magnus Fries
Grubbs II	second-generation Grubbs catalyst
GSH	glutathione
HMPA	Hexamethylphosphoramide
hν	quantum of electromagnetic radiation
IBX	2-Iodoxybenzoic acid
Im	imidazole
IPNBSH	N-Isopropylidene-N′-2-nitrobenzenesulfonyl hydrazine
*i*Pr	isopropyl
HMDS	bis(trimethylsilyl)amide
Lat.	from Latin
LDA	lithium diisopropylamide
LSD	lysergic acid diethylamide
LTMP	lithium 2,2,6,6-tetramethylpiperidide
MDM	trisiloxane, octamethyl-
Me	methyl
MRSA	methicyllin-resistant Staphylococcus aureus
Ms	mesylate
NAD+/NADH	nicotinamide adenine dinucleotide (oxidized and reduced forms)
*n*Bu	*n*-Buthyl
NCS	N-Chlorosuccinimide
Ni_Raney_	Raney catalyst
NIS	N-Iodosuccinimide
NMO	N-Methylmorpholine N-oxide
NMP	N-Methyl-2-pyrrolidone
Ns (pNs)	nosyl, 4-Nitrobenzenesulfonyl
P-450	cytochrome, superfamily of enzymes containing heme that functions as monooxygenases
Pfb	perfluorobutane
PG	protecting group
Ph	phenyl
Piv	pivalic
PNB	p-nitrobenzyl
PS (lipase)	*Pseudomonas*
PTSA	p-Toluenesulfonic acid, TsOH
Py/Pyr	pyridine
R	any functional group/further part of molecules
rt	room temperature
SAM	S-adenosyl methionine
SDR	short-chain dehydrogenases/reductases
Sia	1,2-dimethylpropyl
TBAF	tetra-*n*-butylammonium fluoride
TBDMS	*tert*-Butyldimethylsilyl
TBHP	*tert*-Butyl hydroperoxide
*t*Bu	*tert*-butyl
TEMPO	2,2,6,6-Tetramethylpiperidine 1-oxyl
Tf	triflate
TFA	trifluoroacetic acid
TFE	trifluoroethyl alcohol
THF	tetrahydrofuran
TIPS	triisopropylsilane
TMS	tetramethylsilane
TosMIC	toluenesulfonylmethyl isocyanide
Ts	tosyl, toluenesulfonyl
Tul.	short name of discoverer (botanist) Louis Rene Edmond Tulasne
USA	United States of America
Var.	lat. *Varietas*, variety
Δ	gr. *Delta*, rise of temperature
μ	mikro (prefix)

## References

[1] Ochodzki P., National Research Institute (2015). Zapobieganie Zagrożeniom Związanym z Występowaniem w Ziarnie Zbóż Alkaloidów Pasożytniczego Grzyba Buławinki Czerwonej (Sporysz)—Prevention of Risks Associated with the Presence of Alkaloids in the Cereal Grain Parasitic Fungus of Ergot.

[2] Schiff P.L. (2006). Ergot and Its Alkaloids. Am. J. Pharm. Educ..

[3] Smakosz A., Kurzyna W., Rudko M., Dąsal M. (2021). The Usage of Ergot (Claviceps Purpurea (Fr.) Tul.) in Obstetrics and Gynecology: A Historical Perspective. Toxins.

[4] Krska R., Crews C. (2008). Significance, Chemistry and Determination of Ergot Alkaloids: A Review. Food Addit. Contam. Part A.

[5] Berde B., Schild H.O. (1978). Ergot Alkaloids and Related Compounds.

[6] Stoll A., Petrzilka T., Rutschmann J., Hofmann A., Günthard H.H. (1954). Über Die Stereochemie Der Lysergsäuren Und Der Dihydro-Lysergsäuren. 37. Mitteilung Über Mutterkornalkaloide. Helv. Chim. Acta.

[7] Hofmann A., Ott H., Griot R., Stadler P.A., Frey A.J. (1963). Die Synthese Und Stereochemie Des Ergotamins. (58. Mitteilung Über Mutterkornalkaloide). Helv. Chim. Acta.

[8] Flieger M., Wurst M., Shelby R. (1997). Ergot Alkaloids—Sources, Structures and Analytical Methods. Folia Microbiol..

[9] Gerhards N., Neubauer L., Tudzynski P., Li S.-M. (2014). Biosynthetic Pathways of Ergot Alkaloids. Toxins.

[10] Matławska I. (2008). Farmakognozja.

[11] Seeman P. (2010). Historical Overview: Introduction to the Dopamine Receptors. The Dopamine Receptors.

[12] Pucadyil T.J., Kalipatnapu S., Chattopadhyay A. (2005). The Serotonin1A A Receptor: A Representative Member of the Serotonin Receptor Family. Cell. Mol. Neurobiol..

[13] Halberstadt A.L., Geyer M.A. (2011). Multiple Receptors Contribute to the Behavioral Effects of Indoleamine Hallucinogens. Neuropharmacology.

[14] Nichols D.E. (2016). Psychedelics. Pharmacol. Rev..

[15] Watts V.J., Mailman R.B., Lawler C.P., Neve K.A., Nichols D.E. (1995). LSD and Structural Analogs: Pharmacological Evaluation at D1 Dopamine Receptors. Psychopharmacology.

[16] Bijoch Ł., Pękała M., Beroun A. (2021). Molekularne Podstawy Działania Wybranych Substancji Psychoaktywnych. Postepy Biochem..

[17] Fuentes J.J., Fonseca F., Elices M., Farré M., Torrens M. (2020). Therapeutic Use of LSD in Psychiatry: A Systematic Review of Randomized-Controlled Clinical Trials. Front. Psychiatry.

[18] Smith D.E., Raswyck G.E., Dickerson Davidson L. (2014). From Hofmann to the Haight Ashbury, and into the Future: The Past and Potential of Lysergic Acid Diethlyamide. J. Psychoact. Drugs.

[19] de Groot A.N., van Dongen P.W.J., Vree T.B., Hekster Y.A., van Roosmalen J. (1998). Ergot Alkaloids. Drugs.

[20] Johnson J.W., Ellis M.J., Piquette Z.A., MacNair C., Carfrae L., Bhando T., Ritchie N.E., Saliba P., Brown E.D., Magolan J. (2022). Antibacterial Activity of Metergoline Analogues: Revisiting the Ergot Alkaloid Scaffold for Antibiotic Discovery. ACS Med. Chem. Lett..

[21] Webster J. (1999). Dopamine Agonist Therapy in Hyperprolactinemia. J. Reprod. Med..

[22] Hamon M., Mallat M., Herbet A., Nelson D.L., Audinot M., Pichat L., Glowinski J. (1981). [3 H]Metergoline: A New Ligand of Serotonin Receptors in the Rat Brain. J. Neurochem..

[23] Miller K.J., King A., Demchyshyn L., Niznik H., Teitler M. (1992). Agonist Activity of Sumatriptan and Metergoline at the Human 5-HT1Dβ Receptor: Further Evidence for a Role of the 5-HT1D Receptor in the Action of Sumatriptan. Eur. J. Pharmacol. Mol. Pharmacol..

[24] Hofmann A. (1978). Historical View on Ergot Alkaloids. Pharmacology.

[25] Barger G., Carr F.H. (1907). The Alkaloids of Ergot. J. Chem. Soc. Trans..

[26] Barger G., Dale H.H. (1907). Ergotoxine and Some Other Constituents of Ergot. Biochem. J..

[27] Stoll A. (1918). Zur Kenntnis Der Mutterkornalkaloide. Verh. Schweiz. Nat. Ges..

[28] Rothlin E., Konzett H., Cerletti A. (1954). The antagonism of ergot alkaloids towards the inhibitory response of the isolated rabbit intestine to epinephrine and norepinephrine. J. Pharmacol. Exp. Ther..

[29] Jacobs W.A., Craig L.C. (1935). The structure of the ergot alkaloids. J. Am. Chem. Soc..

[30] Stoll A., Hofmann A., Troxler F. (1949). Über Die Isomerie von Lysergsäure Und Isolysergsäure. 14. Mitteilung Über Mutterkornalkaloide. Helv. Chim. Acta.

[31] Kornfeld E.C., Fornefeld E.J., Kline G.B., Mann M.J., Morrison D.E., Jones R.G., Woodward R.B. (1956). The Total Synthesis of Lysergic Acid. J. Am. Chem. Soc..

[32] Ramage R., Armstrong V.W., Coulton S. (1981). A New Synthetic Route to (±) Lysergic Acid. Tetrahedron.

[33] Stadler P.A., Frey A.J., Troxler F., Hofmann A. (1964). Selektive Reduktions- Und Oxydationsreaktionen an Lysergsäure- Derivaten. 2.3-Dihydro- Und 12-Hydroxy-Lysergsäureamide. 59. Mitteilung Über Mutterkornalkaloide. Helv. Chim. Acta.

[34] Woodward R.B., Hoffmann R. (1969). The Conservation of Orbital Symmetry. Angew. Chem. Int. Ed. Engl..

[35] Stoll A., Hofmann A. (1943). Die Optisch Aktiven Hydrazide Der Lysergsäure Und Der Isolysergsäure. (4. Mitteilung Über Mutterkornalkaloide). Helv. Chim. Acta.

[36] Woodward R.B. (1952). Private Communication between Woodward and Ramage. Ph.D. Thesis.

[37] Křen V., Cvak V. (1999). Ergot The Genus Claviceps (Medicinal and Aromatic Plants—Industrial Profiles).

[38] Martínková L., Křen V., Cvak L., Ovesná M., Přepechalová I. (2000). Hydrolysis of Lysergamide to Lysergic Acid by Rhodococcus Equi A4. J. Biotechnol..

[39] Pauling L. (1942). The Nature of the Chemical Bond.

[40] Blount B.K., Robinson R. (1931). CCCCXXXVI.—Strychnine and Brucine. Part XIII. Note on Dihydroindolylpropionic and Dihydroindolylbutyric Acids. J. Chem. Soc..

[41] Uhle F.C. (1949). The Synthesis of 5-Keto-1,3,4,5-Tetrahydrobenz[Cd]Indole. A Synthesis of 4-Substituted Indoles. J. Am. Chem. Soc..

[42] Johnson W.S., Belew J.S., Chinn L.J., Hunt R.H. (1953). Studies Relating to the Formation and Reactions of Glycidic Esters. J. Am. Chem. Soc..

[43] Price C.C., Krishnamurti I.V. (1950). The Acid-Catalyzed Reaction of Formaldehyde with Allyl Cyanide. J. Am. Chem. Soc..

[44] Kleiderer E.C., Kornfeld E.C. (1948). Raney nickel as an organic oxidation-reduction catalyst. J. Org. Chem..

[45] Stoll A., Hofmann A. (1937). Racemische Lysergsäure Und Ihre Auflösung in Die Optischen Antipoden.-Zweite, Vorläufige Mitteilung Über Mutterkornalkaloide. Hoppe Seyler’s Z. Für Physiol. Chem..

[46] Smith S., Timmis G.M. (1936). A New Alkaloid of Ergot. Nature.

[47] Julia M., Le Goffic F., Igolen J., Baillarge M. (1969). Une Nouvelle Synthese de l’acide Lysergique. Tetrahedron Lett..

[48] House H.O., Rasmussen G.H. (1961). Stereoselective Synthesis of α-Substituted α,β-Unsaturated Esters. J. Org. Chem..

[49] Jackson A.G., Kenner G.W., Moore G.A., Ramage R., Thorpe W.D. (1976). Activation of Carboxylic Acids as Diphenylphosphinic Mixed Anhydrides: Application to Peptide Chemistry. Tetrahedron Lett..

[50] Bu’Lock J.D., Harley-Mason J. (1951). 497. Melanin and Its Precursors. Part III. New Syntheses of 5: 6-Dihydroxyindole and Its Derivatives. J. Chem. Soc..

[51] Troxler F. (1968). Beiträge Zur Chemie Der 6-Methy1-8-Ergolen-8-Carbonsäure. 69. Mitteilung Über Mutterkornalkaloide. Helv. Chim. Acta.

[52] Eschweiler W. (1905). Ersatz von an Stickstoff Gebundenen Wasserstoffatomen Durch Die Methylgruppe Mit Hülfe von Formaldehyd. Ber. Dtsch. Chem. Ges..

[53] Clarke H.T., Gillespie H.B., Weisshaus S.Z. (1933). The Action of Formaldehyde on Amines and Amino Acids 1. J. Am. Chem. Soc..

[54] Fehr T., Stadler P.A., Hofmann A. (1970). Demethylierung Des Lysergsäuregerüstes. 73. Mitteilung Über Mutterkornalkaloide. Helv. Chim. Acta.

[55] Rebek J., Tai D.F. (1983). A New Synthesis of Lysergic Acid. Tetrahedron Lett..

[56] Rebek J., Tai D.F., Shue Y.K. (1984). Synthesis of Ergot Alkaloids from Tryptophan. J. Am. Chem. Soc..

[57] Hanessian S. (1967). Selective Hydrolysis of Amide Bonds in Acetamido Deoxy Sugars.-Ethyl Acetamidium Fluoroborates. Tetrahedron Lett..

[58] Smith S., Timmis G.M. (1936). 311. The Alkaloids of Ergot. Part VII. IsoErgine and Isolysergic Acids. J. Chem. Soc..

[59] Ninomiya I., Kiguchi T., Hashimoto C., Naito T. (1982). A New Synthesis of (±)-Lysergic Acid. Heterocycles.

[60] Ninomiya I., Naito T. (1984). Enamide Cyclization and Its Application to the Synthesis of Heterocycles. J. Synth. Org. Chem. Japan.

[61] Nichols D.E., Robinson J.M., Li G.S., Cassady J.M., Floss H.G. (1977). An improved synthesis of 1-benzoyl-4-keto-1,2,2a,3,4,5-hexahydrobenz [cd]indole. Org. Prep. Proced. Int..

[62] Ninomiya I., Hashimoto C., Kiguchi T., Naito T. (1985). Photocyclisation of Enamides. Part 24. Total Synthesis of (±)-Isofumigaclavine B and (±)-Lysergic Acid. J. Chem. Soc. Perkin Trans. 1.

[63] Tasker N.R., Wipf P. (2022). A Short Synthesis of Ergot Alkaloids and Evaluation of the 5-HT 1/2 Receptor Selectivity of Lysergols and Isolysergols. Org. Lett..

[64] Hendrickson J.B., Wang J. (2004). A New Synthesis of Lysergic Acid. Org. Lett..

[65] Doll M.K.-H. (1999). A Short Synthesis of the 8-Azaergoline Ring System by Intramolecular Tandem Decarboxylation−Cyclization of the Minisci-Type Reaction. J. Org. Chem..

[66] Bekkam M., Mo H., Nichols D.E. (2012). A Reported “New Synthesis of Lysergic Acid” Yields Only The Derailment Product: Methyl 5-Methoxy-4,5-Dihydroindolo[4,3-f,g ]Quinoline-9-Carboxylate. Org. Lett..

[67] Zhao Y. (2010). Reduction of Tertiary Benzamides to Benzaldehydes by an in Situ–Generated Schwartz Reagent (Cp2Zr(H)Cl).

[68] Points G.L., Stout K.T., Beaudry C.M. (2020). Regioselective Formation of Substituted Indoles: Formal Synthesis of Lysergic Acid. Chem. Eur. J..

[69] Zhao Y., Snieckus V. (2014). A Practical in Situ Generation of the Schwartz Reagent. Reduction of Tertiary Amides to Aldehydes and Hydrozirconation. Org. Lett..

[70] Moldvai I., Temesvári-Major E., Incze M., Szentirmay É., Gács-Baitz E., Szántay C. (2004). Enantioefficient Synthesis of α-Ergocryptine: First Direct Synthesis of (+)-Lysergic Acid. J. Org. Chem..

[71] Teranishi K., Hayashi S., Nakatsuka S., Goto T. (1994). A Facile Biomimetic Synthesis of Uhle’s Ketone by the Regioselective Friedel-Crafts Cyclization of Indole-3-Ylpropionyl Chloride. Tetrahedron Lett..

[72] Roth M., Dubs P., Götschi E., Eschenmoser A. (1971). Sulfidkontraktion via Alkylative Kupplung: Eine Methode Zur Darstellung von β-Dicarbonylderivaten. Über Synthetische Methoden, 1. Mitteilung. Helv. Chim. Acta.

[73] Moldvai I., Temesvári-Major E., Incze M., Dörnyei G., Szentirmay É., Szántay C. (2005). Synthetic Route to Ergot Alkaloids. Helv. Chim. Acta.

[74] Rathnayake U., Garner P. (2021). Asymmetric Synthesis of Lysergic Acid via an Intramolecular (3+2) Dipolar Cycloaddition/Ring-Expansion Sequence. Org. Lett..

[75] Zuo Z., Ma D. (2011). Enantioselective Total Syntheses of Communesins A and B. Angew. Chem. Int. Ed. Engl..

[76] Métro T.-X., Appenzeller J., Gomez Pardo D., Cossy J. (2006). Highly Enantioselective Synthesis of β-Amino Alcohols. Org. Lett..

[77] Jarvis S.B.D., Charette A.B. (2011). Synthesis of Enantiopure Substituted Piperidines via an Aziridinium Ring Expansion. Org. Lett..

[78] Oestreich M. (2009). The Mizoroki–Heck Reaction.

[79] Cacchi S., Giuseppe Ciattini P., Morera E., Ortar G. (1988). A Concise, Palladium-Catalyzed Approach to (±)-Lysergic Acid. Tetrahedron Lett..

[80] Kurihara T., Terada T., Yoneda R. (1986). A New Synthesis of (±)-Lysergic Acid. Chem. Pharm. Bull..

[81] Kurihara T., Terada T., Harusawa S., Yoneda R. (1987). Synthetic Studies of (±)-Lysergic Acid and Related Compounds. Chem. Pharm. Bull..

[82] Busqué F., de March P., Figueredo M., Font J., Gallagher T., Milán S. (2002). Efficient Synthesis of (S)-3,4-Dihydro-2-Pivaloyloxymethyl-2H-Pyrrole 1-Oxide. Tetrahedron Asymmetry.

[83] Birkofer L., Bierwirth E., Ritter A. (1961). Decarbobenzoxylierungen Mit Triäthylsilan. Chem. Ber..

[84] Ghosez L., Haveaux B., Viehe H.G. (1969). Alkyl and Arylα-Chloro Enamines. Angew. Chem. Int. Ed. Engl..

[85] Oppolzer W., Francotte E., Bättig K. (1981). Total Synthesis of (±)-Lysergic Acid by an Intramolecular Imino- Diels—Alder Reaction. Preliminary Communication. Helv. Chim. Acta.

[86] Kurokawa T., Isomura M., Tokuyama H., Fukuyama T. (2009). Synthesis of Lysergic Acid Methyl Ester via the Double Cyclization Strategy. Synlett.

[87] Ninomiya I., Hashimoto C., Kiguchi T., Naito T., Barton D.H.R., Lusinchi X., Milliet P. (1990). Dehydrogenation with Benzeneseleninic Anhydride in the Total Synthesis of Ergot Alkaloids. J. Chem. Soc. Perkin Trans. 1.

[88] Umezaki S., Yokoshima S., Fukuyama T. (2013). Total Synthesis of Lysergic Acid. Org. Lett..

[89] Crimmins M.T., King B.W., Tabet E.A., Chaudhary K. (2001). Asymmetric Aldol Additions: Use of Titanium Tetrachloride and (−)-Sparteine for the Soft Enolization of N-Acyl Oxazolidinones, Oxazolidinethiones, and Thiazolidinethiones. J. Org. Chem..

[90] Scholl M., Ding S., Lee C.W., Grubbs R.H. (1999). Synthesis and Activity of a New Generation of Ruthenium-Based Olefin Metathesis Catalysts Coordinated with 1,3-Dimesityl-4,5-Dihydroimidazol-2-Ylidene Ligands. Org. Lett..

[91] Kanno R., Yokoshima S., Kanai M., Fukuyama T. (2018). Total Synthesis of (+)-Lysergic Acid. J. Antibiot..

[92] Evans D.A., Ennis M.D., Mathre D.J. (1982). Asymmetric Alkylation Reactions of Chiral Imide Enolates. A Practical Approach to the Enantioselective Synthesis of Alpha.-Substituted Carboxylic Acid Derivatives. J. Am. Chem. Soc..

[93] Liu Q., Jia Y. (2011). Total Synthesis of (+)-Lysergic Acid. Org. Lett..

[94] Liu Q., Zhang Y.-A., Xu P., Jia Y. (2013). Total Synthesis of (+)-Lysergic Acid. J. Org. Chem..

[95] González-Gómez J.C., Medjahdi M., Foubelo F., Yus M. (2010). Stereoselective α-Aminoallylation of Aldehydes with Chiral Tert-Butanesulfinamides and Allyl Bromides. J. Org. Chem..

[96] Oppolzer W., Grayson J.I. (1980). Stereoselective Total Syntheses of (±)-Chanoclavine I and (±)-Isochanoclavine I by an Intramolecular Nitrone-Olefin/Cycloaddition. Preliminary Communication. Helv. Chim. Acta.

[97] Plieninger H., Wagner C., Immel H. (1971). Synthese von 4-[E-4-Hydroxy-3-Methyl-Δ2-Butenyl]-Tryptophan-[4-14C] Sowie -Tryptamin-[4-14C] Und Einbau in Clavin-Alkaloide. Justus Liebigs Ann. Chem..

[98] Inuki S., Oishi S., Fujii N., Ohno H. (2008). Total Synthesis of (±)-Lysergic Acid, Lysergol, and Isolysergol by Palladium-Catalyzed Domino Cyclization of Amino Allenes Bearing a Bromoindolyl Group. Org. Lett..

[99] Iwata A., Inuki S., Oishi S., Fujii N., Ohno H. (2011). Formal Total Synthesis of (+)-Lysergic Acid via Zinc(II)-Mediated Regioselective Ring-Opening Reduction of 2-Alkynyl-3-Indolyloxirane. J. Org. Chem..

[100] Inuki S., Iwata A., Oishi S., Fujii N., Ohno H. (2011). Enantioselective Total Synthesis of (+)-Lysergic Acid, (+)-Lysergol, and (+)-Isolysergol by Palladium-Catalyzed Domino Cyclization of Allenes Bearing Amino and Bromoindolyl Groups. J. Org. Chem..

[101] Kimura M., Futamata M., Mukai R., Tamaru Y. (2005). Pd-Catalyzed C3-Selective Allylation of Indoles with Allyl Alcohols Promoted by Triethylborane. J. Am. Chem. Soc..

[102] Hofmeister H., Annen K., Laurent H., Wiechert R. (1984). A Novel Entry to 17α-Bromo-and 17α-Iodoethynyl Steroids. Angew. Chem. Int. Ed. Engl..

[103] Myers A.G., Zheng B. (1996). New and Stereospecific Synthesis of Allenes in a Single Step from Propargylic Alcohols. J. Am. Chem. Soc..

[104] Lauchli R., Shea K.J. (2006). A Synthesis of the Welwistatin Core. Org. Lett..

[105] Uenishi J., Kawahama R., Shiga Y., Yonemitsu O., Tsuji J. (1996). A General and Convenient Synthetic Method of Geometrically Pure (Z)-1-Bromo-1-Alkenes. Tetrahedron Lett..

[106] Movassaghi M., Ahmad O.K. (2007). N -Isopropylidene- N’-2-Nitrobenzenesulfonyl Hydrazine, a Reagent for Reduction of Alcohols via the Corresponding Monoalkyl Diazenes. J. Org. Chem..

[107] Marino J.P., Osterhout M.H., Padwa A. (1995). An Approach to Lysergic Acid Utilizing an Intramolecular Isomuenchnone Cycloaddition Pathway. J. Org. Chem..

[108] Padwa A., Austin D.J. (1994). Ligand Effects on the Chemoselectivity of Transition Metal Catalyzed Reactions Ofα-Diazo Carbonyl Compounds. Angew. Chem. Int. Ed. Engl..

[109] Barton D.H.R., McCombie S.W. (1975). A New Method for the Deoxygenation of Secondary Alcohols. J. Chem. Soc. Perkin Trans. 1.

[110] Parsons P.J., Penkett C.S., Shell A.J. (1996). Tandem Reactions in Organic Synthesis: Novel Strategies for Natural Product Elaboratoin and the Development of New Synthetic Methodology. Chem. Rev..

[111] Cladingboel D.E., Parsons P.J. (1990). Synthesis of Lysergic Acid Derivatives by Triple Radical Cyclisation. J. Chem. Soc. Chem. Commun..

[112] Özlü Y., Cladingboel D.E., Parsons P.J. (1994). Tandem Radical Cyclisations: Synthesis of Lysergic Acid Derivatives. Tetrahedron.

[113] Floss H.G. (1976). Biosynthesis of Ergot Alkaloids and Related Compounds. Tetrahedron.

[114] Mukherjee J., Menge M. (2000). Progress and Prospects of Ergot Alkaloid Research. New Products and New Areas of Bioprocess Engineering. Advances in Biochemical Engineering/Biotechnology.

[115] Tsai H.F., Wang H., Gebler J.C., Poulter C.D., Schardl C.L. (1995). The Claviceps Purpurea Gene Encoding Dimethylallyltryptophan Synthase, the Committed Step for Ergot Alkaloid Biosynthesis. Biochem. Biophys. Res. Commun..

[116] Lee S.-L., Floss H.G., Heinstein P. (1976). Purification and Properties of Dimethylallylpyrophosphate: Tryptophan Dimethylallyl Transferase, the First Enzyme of Ergot Alkaloid Biosynthesis in *Claviceps* Sp. SD 58. Arch. Biochem. Biophys..

[117] Metzger U., Schall C., Zocher G., Unsöld I., Stec E., Li S.-M., Heide L., Stehle T. (2009). The Structure of Dimethylallyl Tryptophan Synthase Reveals a Common Architecture of Aromatic Prenyltransferases in Fungi and Bacteria. Proc. Natl. Acad. Sci. USA.

[118] Rigbers O., Li S.-M. (2008). Ergot Alkaloid Biosynthesis in Aspergillus Fumigatus. J. Biol. Chem..

[119] Tudzynski P., Correia T., Keller U. (2001). Biotechnology and Genetics of Ergot Alkaloids. Appl. Microbiol. Biotechnol..

[120] Lorenz N., OlŠovská J., šulc M., Tudzynski P. (2010). Alkaloid Cluster Gene CcsA of the Ergot Fungus Claviceps Purpurea Encodes Chanoclavine I Synthase, a Flavin Adenine Dinucleotide-Containing Oxidoreductase Mediating the Transformation of N-Methyl-Dimethylallyltryptophan to Chanoclavine I. Appl. Environ. Microbiol..

[121] Goetz K.E., Coyle C.M., Cheng J.Z., O’Connor S.E., Panaccione D.G. (2011). Ergot Cluster-Encoded Catalase Is Required for Synthesis of Chanoclavine-I in Aspergillus Fumigatus. Curr. Genet..

[122] Ryan K., Moore C., Panaccione D. (2013). Partial Reconstruction of the Ergot Alkaloid Pathway by Heterologous Gene Expression in Aspergillus Nidulans. Toxins.

[123] Wallwey C., Matuschek M., Li S.-M. (2010). Ergot Alkaloid Biosynthesis in Aspergillus Fumigatus: Conversion of Chanoclavine-I to Chanoclavine-I Aldehyde Catalyzed by a Short-Chain Alcohol Dehydrogenase FgaDH. Arch. Microbiol..

[124] Cheng J.Z., Coyle C.M., Panaccione D.G., O’Connor S.E. (2010). Controlling a Structural Branch Point in Ergot Alkaloid Biosynthesis. J. Am. Chem. Soc..

[125] Matuschek M., Wallwey C., Xie X., Li S.-M. (2011). New Insights into Ergot Alkaloid Biosynthesis in Claviceps Purpurea: An Agroclavine Synthase EasG Catalyses, via a Non-Enzymatic Adduct with Reduced Glutathione, the Conversion of Chanoclavine-I Aldehyde to Agroclavine. Org. Biomol. Chem..

[126] Kim S.-U., Cho Y.-J., Floss H., Anderson J. (1983). Conversion of Elymoclavine to Paspalic Acid by a Particulate Fraction from an Ergotamine-Producing Strain of *Claviceps* sp.. Planta Med..

[127] Maier W., Schumann B., Gröger D. (1988). Microsomal Oxygenases Involved in Ergoline Alkaloid Biosynthesis of VariousClaviceps Strains. J. Basic Microbiol..

[128] Britton K.N., Steen C.R., Davis K.A., Sampson J.K., Panaccione D.G. (2022). Contribution of a Novel Gene to Lysergic Acid Amide Synthesis in Metarhizium Brunneum. BMC Res. Notes.

[129] Chen J.-J., Han M.-Y., Gong T., Yang J.-L., Zhu P. (2017). Recent Progress in Ergot Alkaloid Research. RSC Adv..

[130] Robinson S.L., Panaccione D.G. (2014). Heterologous Expression of Lysergic Acid and Novel Ergot Alkaloids in Aspergillus Fumigatus. Appl. Environ. Microbiol..

[131] Wong G., Lim L.R., Tan Y.Q., Go M.K., Bell D.J., Freemont P.S., Yew W.S. (2022). Reconstituting the Complete Biosynthesis of D-Lysergic Acid in Yeast. Nat. Commun..

